# Statistical analysis of the count and profitability of air conditioners

**DOI:** 10.1016/j.dib.2018.05.035

**Published:** 2018-05-15

**Authors:** EL Houssainy A. Rady, Salah M. Mohamed, Alaa A. Abd Elmegaly

**Affiliations:** Department of Applied Statistics and Econometrics, Institute of Statistical Studies and Research, Cairo University, Egypt

**Keywords:** Air conditioner, Company, Kolmogorov–Smirnov, Kruskal–Wallis test, Profit, Statistics

## Abstract

This article presents the statistical analysis of the number and profitability of air conditioners in an Egyptian company. Checking the same distribution for each categorical variable has been made using Kruskal–Wallis test.

**Specifications Table**

**Table:** 

Subject area	Economics
More specific subject area	Business Company, Social Statistics
Type of data	Table and text file
How data was acquired	Collected by the author
Data format	Raw and partially analyzed (Descriptive and Inferential)
Experimental factors	Data sets on devices sold in a different
	Types of air conditioners in an Egyptian Company
Experimental features	Observations on the number of air conditioners that sold in the company for six different types of air conditioners and its profits.
Data source location	The data was obtained from one of the air conditioner company in Egypt
Data accessibility	All the data are available this data article

**Value of the data**

• Data are useful in calculating the appropriate quantities of each type of air conditioner.
• The data could be used as one of vital tools in assessing air conditioners companies competitiveness.
• Data analysis can be useful in detecting more and fewer types of demand by consumers.
• Data can be useful in identifying the most profitable species in the organization.
• Data can be used to monitor compliance with the decisions and strategy necessary to determine the price of air conditioning.
• Data can be expanded to include behavioral attitudes and customer preferences types of air conditioners.

## 1 Data

This is a simple data set that summarizes the performance of a small AC company who went out of business shortly after March 2013. Considering this is a small business that eventually failed. The data in this article represent 1058 units of air conditioner that sailed from July 2007 to March 2013 in an Egyptian company called Pure technology, we decomposed these units as The ISM frequency data on traditional vs. modern views is used, that found in Hunter and Takane [1], the data were as follows ( Table 1):

**Table 1:** The count of sales units of air conditioner at different cases.

Nos.	Sex	Cordon	Season	1.5 HP/b	2.25 HP/b	3Hp/b	1.5 Hp/c	2.25 HP/c	3HP/c	Total
1	M	Y	Summer	17	6	13	52	32	26	146
2	M	Y	Winter	3	0	0	3	1	2	9
3	M	Y	Autumn	0	0	1	12	6	3	22
4	M	Y	Spring	30	15	7	47	21	21	141
5	F	Y	Summer	6	1	5	6	6	1	25
6	F	Y	Autumn	0	0	0	0	3	1	4
7	F	Y	Spring	1	0	1	4	0	3	9
8	C	Y	Summer	0	0	0	0	2	6	8
9	C	Y	Winter	0	0	0	2	0	1	3
10	C	Y	Autumn	4	0	0	1	4	6	15
11	C	Y	Spring	5	0	1	2	4	16	28
12	M	N	Summer	20	15	11	29	26	29	130
13	M	N	Winter	1	2	2	3	0	1	9
14	M	N	Autumn	14	9	5	17	9	10	64
15	M	N	Spring	45	13	11	37	29	21	156
16	F	N	Summer	2	0	1	2	3	3	11
17	F	N	Winter	0	0	1	4	3	1	9
18	F	N	Autumn	1	1	1	5	1	3	12
19	F	N	Spring	0	1	0	2	3	3	9
20	C	N	Summer	2	1	2	1	8	28	42
21	C	N	Winter	3	1	8	2	5	16	35
22	C	N	Autumn	21	2	2	7	11	8	51
23	C	N	Spring	9	5	4	12	28	62	120
				184	72	76	250	205	271	1058

The author collected the data from an Egyptian air conditioner Company called Pure Technology. Where we make the cases constrained (G) is:

1. Sex of the client (M=Male, F=Female and C=company)
2. Cordon (the where that the client live) of the client (Y=Yes and N=No)
3. Season of the sale (summer, winter, autumn and spring).

In addition, the variables constrained (H) is:

1. 1.5 HP/b represent the air condition with power 1.5 horse and it is hot and cold
2. 2.25 HP/b represent the air condition with power 2.25 horse and it is hot and cold
3. 3HP/b represent the air condition with power 3 horse and it is hot and cold
4. 1.5 HP/c represent the air condition with power 1.5 horse and it is cold
5. 2.25 HP/c represent the air condition with power 2.25 horse and it is cold
6. 3 HP/c represent the air condition with power 3 horse and it is cold

Moreover, the matrix G was as follows (Table 2):

**Table 2:** The cases constrained matrix G.

M	F	C	Y	N	Summer	Winter	Autumn	Spring
G1	G2	G3	G4	G5	G6	G7	G8	G9
1	0	0	1	0	1	0	0	0
1	0	0	1	0	0	1	0	0
1	0	0	1	0	0	0	1	0
1	0	0	1	0	0	0	0	1
0	1	0	1	0	1	0	0	0
0	1	0	1	0	0	0	1	0
0	1	0	1	0	0	0	0	1
0	0	1	1	0	1	0	0	0
0	0	1	1	0	0	1	0	0
0	0	1	1	0	0	0	1	0
0	0	1	1	0	0	0	0	1
1	0	0	0	1	1	0	0	0
1	0	0	0	1	0	1	0	0
1	0	0	0	1	0	0	1	0
1	0	0	0	1	0	0	0	1
0	1	0	0	1	1	0	0	0
0	1	0	0	1	0	1	0	0
0	1	0	0	1	0	0	1	0
0	1	0	0	1	0	0	0	1
0	0	1	0	1	1	0	0	0
0	0	1	0	1	0	1	0	0
0	0	1	0	1	0	0	1	0
0	0	1	0	1	0	0	0	1

(The data represent the constrained that found in cases, we get it from Table 1)

where:

G1=1 if sex=M, G1=0 otherwise

G2=1 if sex=F, G2=0 otherwise

G3=1 if sex=C, G3=0 otherwise

G4=1 if Cordon=Y, G4=0 otherwise

G5=1 if Cordon=N, G5=0 otherwise

G6=1 if Season= summer, G6=0 otherwise

G7=1 if Season= winter, G7=0 otherwise

G8=1 if Season= autumn, G8=0 otherwise

G9=1 if Season= spring, G9=0 otherwise

The column constrained was making by combining between the power of the unit measuring by **HP** and kind of this unit (cold only or cold and hot) and the matrix **H** was as follows (Table 3):

**Table 3:** The variables constrained matrix H.

	1.5 HP	2.25Hp	3Hp	b	c
	H1	H2	H3	H4	H5
1.5 HP/b	1	0	0	1	0
2.25 HP/b	0	1	0	1	0
3Hp/b	0	0	1	1	0
1.5Hp/c	1	0	0	0	1
2.25 HP/c	0	1	0	0	1
3 HP/c	0	0	1	0	1

(The data represent the constrained that found in variables, we get it from Table 1).

The H matrix represent combination between (1.5 HP, 2.25HP, 3HP) and the type of air conditioner (b, c). For example for the air conditioner, 1.5HP/b it takes 1 at the column 1.5HP and the column b. otherwise it takes 0

In addition, the next table indicate the profit of the sales units of air conditioner at different cases (Table 4).

**Table 4:** The profit of the sales units of air conditioner at different cases (all values with Egyptian pound EGP.

Nos.	Sex	Cordon	Season	1.5 HP/b	2.25 HP/b	3Hp/b	1.5Hp/c	2.25 HP/c	3 HP/c	Total
1	M	Y	Summer	6223	2474	5440	16947	11767	9918	52769
2	M	Y	Winter	1050	0	0	335	210	849	2444
3	M	Y	Autumn	0	0	440	4149	2055	1230	7874
4	M	Y	Spring	11120	6040	2739	16161	7222	8461	51743
5	F	Y	Summer	2124	449	2260	2000	1760	352	8945
6	F	Y	Autumn	0	0	0	0	1150	410	1560
7	F	Y	Spring	400	0	440	1399	0	1215	3454
8	C	Y	Summer	0	0	0	0	188.31	2430	2618.31
9	C	Y	Winter	0	0	0	−45	0	−123	−168
10	C	Y	Autumn	−325	0	0	−75	4080	2176.68	5856.68
11	C	Y	Spring	1265	0	440	350	1025	6560	9640
12	M	N	Summer	7449	6094	3819	9323	9704	10608	46997
13	M	N	Winter	450	898	849	1050	0	410	3657
14	M	N	Autumn	5025	3252	2010	6025	2960	3461	22733
15	M	N	Spring	9314	4390	4555	13214	10460	8516	50449
16	F	N	Summer	1450	0	455	435	895	1230	4465
17	F	N	Winter	0	0	440	1132	1199	449	3220
18	F	N	Autumn	375	405	440	2150	210	1302	4882
19	F	N	Spring	0	405	0	625	655	1315	3000
20	C	N	Summer	1175	405	880	350	3105	10767	16682
21	C	N	Winter	1050	455	3060	330	2195	6560	13650
22	C	N	Autumn	2689	834	153.2	1987	2813	3228	11704.2
23	C	N	Spring	889	1637	255	4116	5825	9510	22232
				51723	27738	28675	81958	69478.3	90834.7	350407.19

(Collected from an Egyptian air conditioner Company called Pure Technology).

Descriptive statistics was used to summarize the data and to provide plots for proper visualization and understanding. SPSS version 24 and Excel version 2013 were used for the analyses in this paper. The data set is summarized in Table 5.

**Table 5:** Summary statistics of the dataset (count of air conditioner).

Type	1.5 HP/b	2.25 HP/b	3Hp/b	1.5 Hp/c	2.25 HP/c	3HP/c
Mean	8	3.13	3.30	10.87	8.91	11.78
Min	0	0	0	0	0	1
Max	45	15	13	52	32	62
Sum	184	72	76	250	205	271

**Fig. 1:**
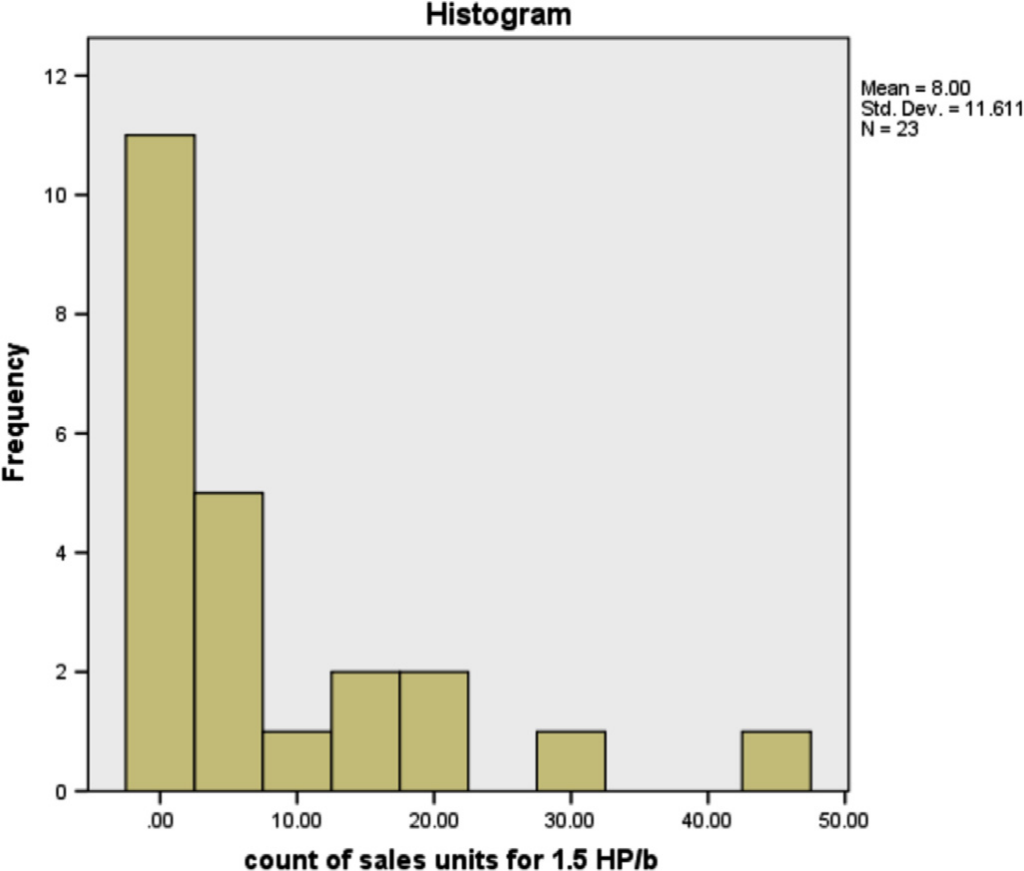
The histogram for the air conditioner type 1.5 HP/b.

**Fig. 2:**
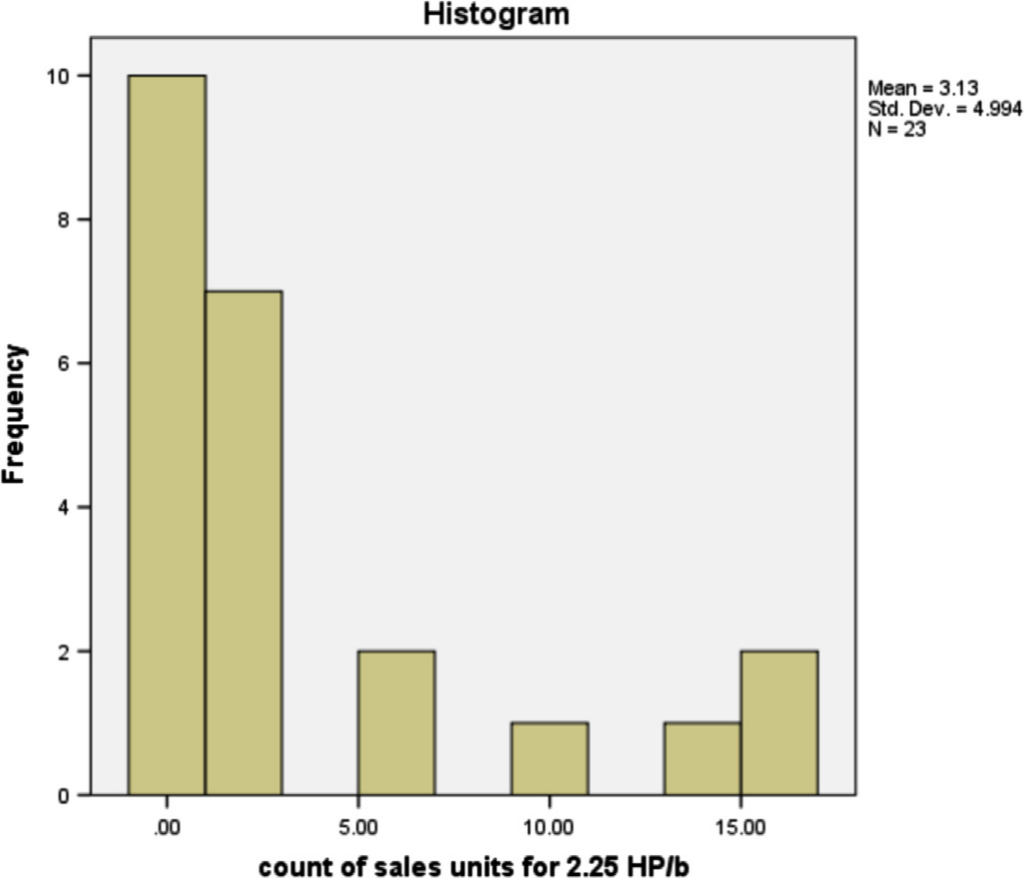
The histogram for the air conditioner type 2.25 HP/b.

**Fig. 3:**
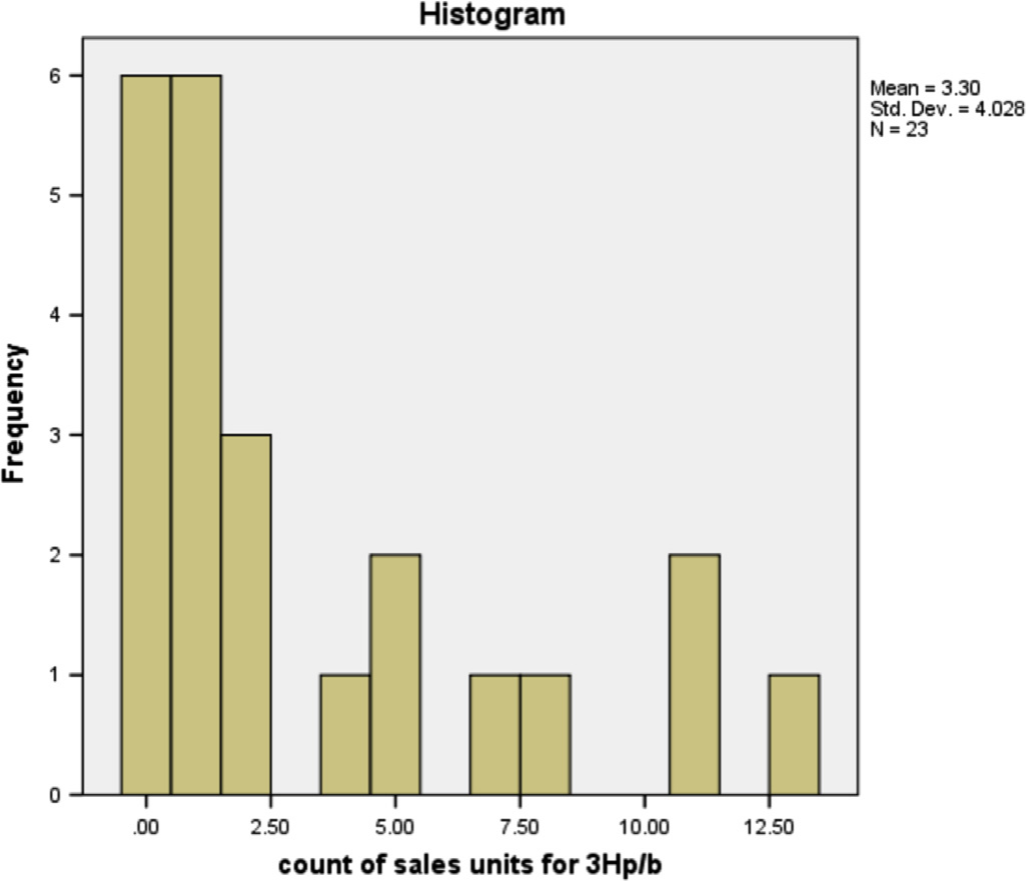
The histogram for the air conditioner type 3 HP/b.

**Fig. 4:**
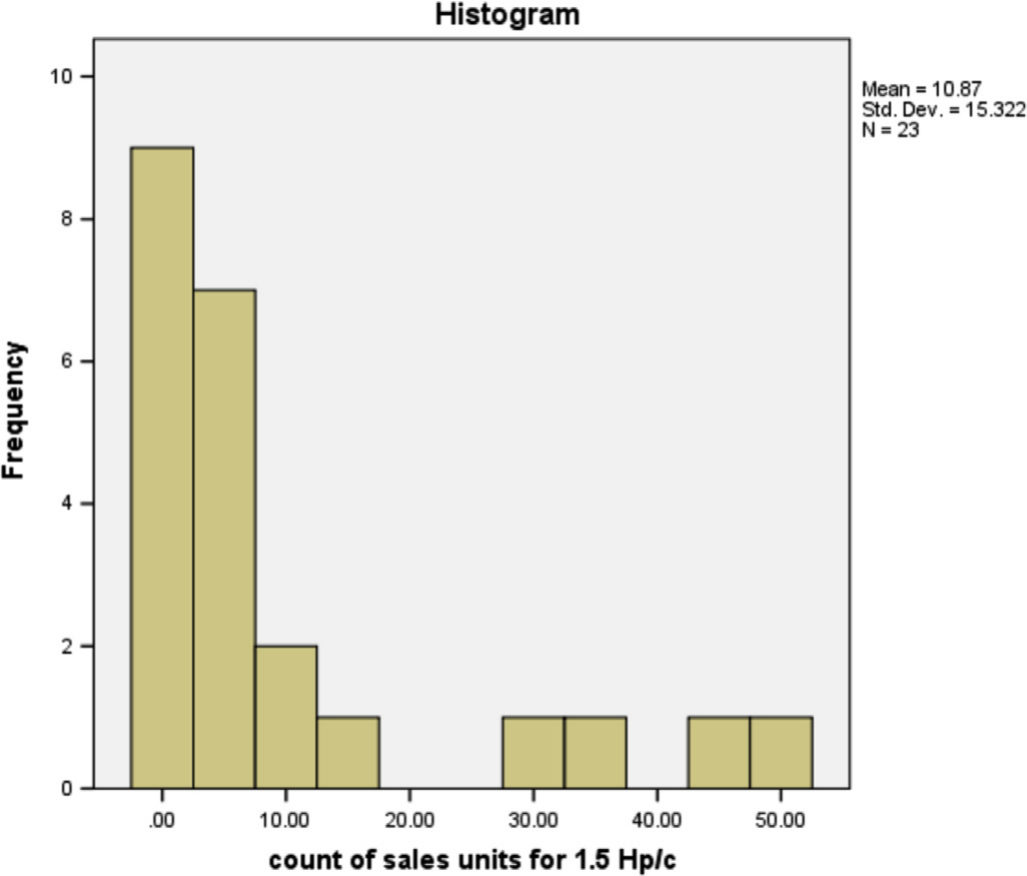
The histogram for the air conditioner type 1.5 HP/c.

**Fig. 5:**
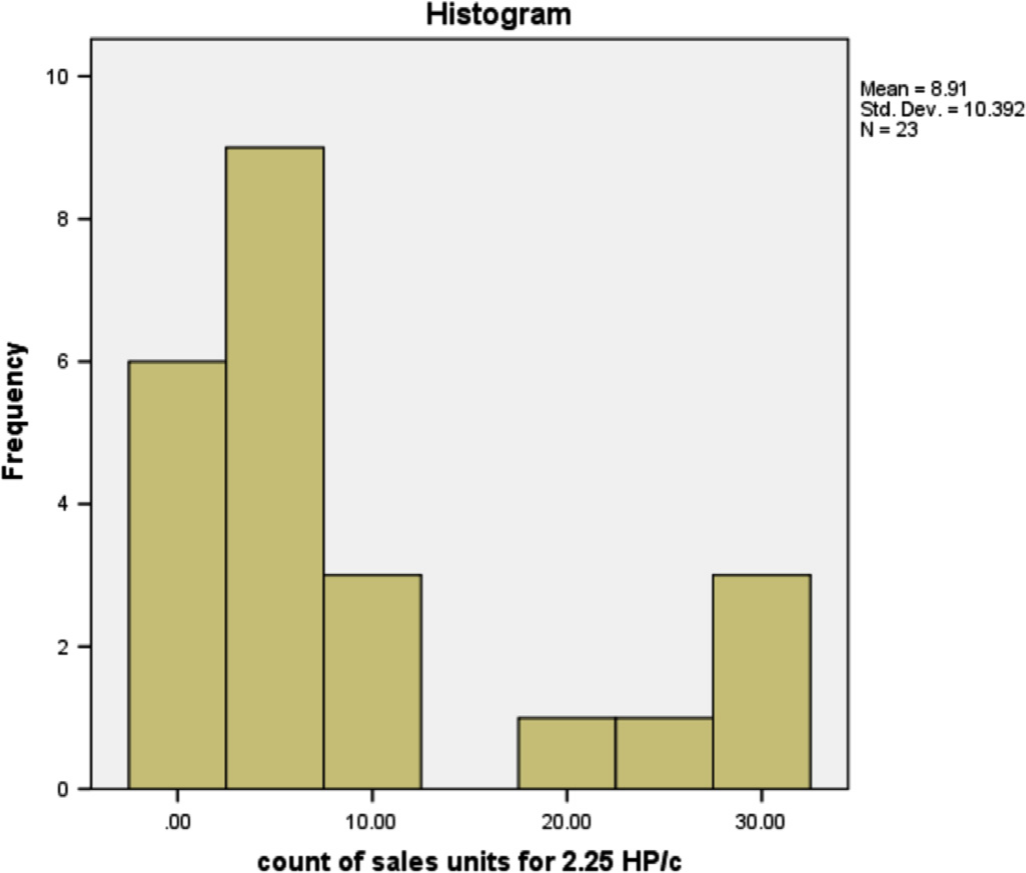
The histogram for the air conditioner type 2.25 HP/c.

**Fig. 6:**
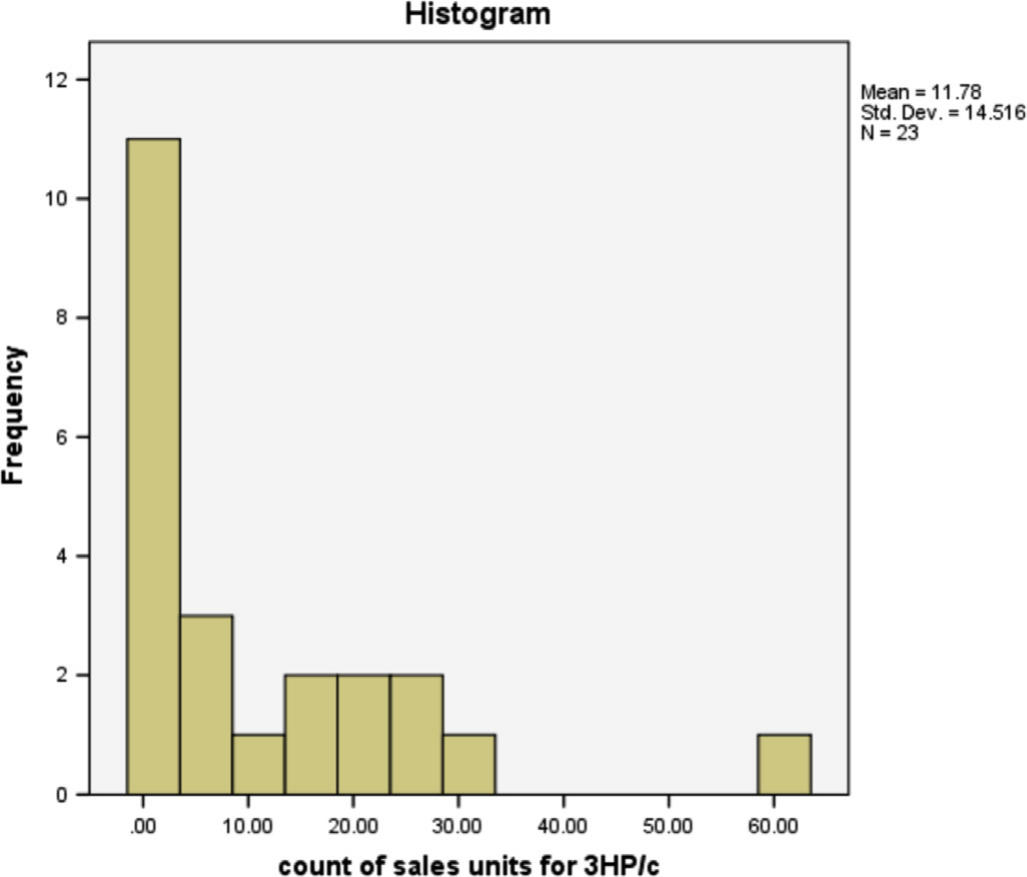
The histogram for the air conditioner type 3 HP/c.

The information in Table 5 shows that more people prefer the 3HP/c air conditioner that has the most sales of any other type of air conditioner. The type of air conditioner with the highest sold units is 3HP/c, although the number of users of this type of air conditioner is not the highest, but on average, customers purchased as many units of this type. This is reasonable because, in the true sense, existing air-conditioner users can be either personal, business or companies. The sold units patterns for all air conditioner types are provided in form of histogram in Fig. 1, Fig. 2, Fig. 3, Fig. 4, Fig. 5, Fig. 6 respectively.

**Fig. 7:**
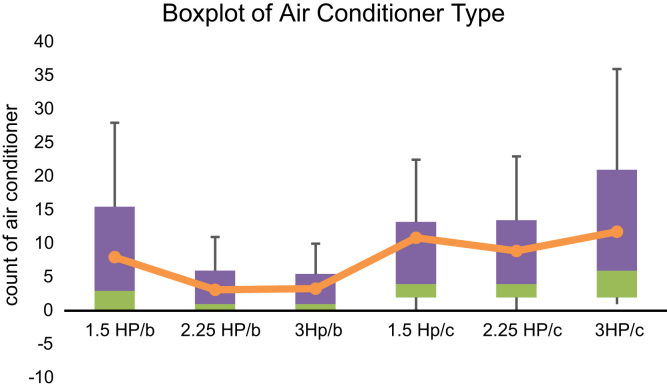
A Boxplot representing the data set.

In addition, the boxplot representing the mean amount of sales in the various air conditioners types is displayed in Fig. 7.

The impact of the current air conditioner is also being identified in the plot provided in Fig. 7. The mean count in each air conditioner type with their respective 95% Confidence Interval (C.I) is displayed in Table 6.

**Table 6:** 95% confidence interval for the mean.

**Type**	**1.5 HP/b**	**2.25 HP/b**	**3Hp/b**	**1.5 Hp/c**	**2.25 HP/c**	**3HP/c**
**Mean**	8.00	3.13	3.30	10.87	8.91	11.78
**sd**	11.36	4.88	3.94	14.98	10.16	14.20
**Upper Limit**	30.26	12.70	11.02	40.24	28.83	39.61
**Lower Limit**	−14.26	−6.44	−4.42	−18.50	−11.01	−16.04

**Fig. 8:**
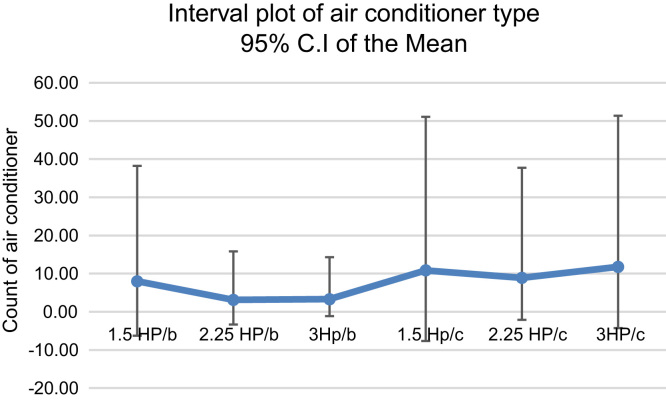
A plot for the 95% C.I for the mean count of air conditioner.

The 95% confidence interval plot for the mean of the amount deposited in the various air conditioner types is displayed in Fig. 8.

### 1.1 Checking the normality distribution of the data

Kolmogorov–Smirnov test is used to check the normality distribution of the data. Where the null hypothesis refer to the count of air conditioner is distributed normally versus the alternative hypothesis that refer to the count of air conditioner is not distributed normally. Table 7 indicates the results as follows:

**Table 7:** Check the normality distribution of the data.

	Kolmogorov–Smirnov^a^			Shapiro–Wilk		
	Statistic	df	Sig.	Statistic	df	Sig.
1.5 HP/b	0.264	23	0.000	0.726	23	0.000
2.25 HP/b	0.329	23	0.000	0.667	23	0.000
3Hp/b	0.279	23	0.000	0.788	23	0.000
1.5 Hp/c	0.295	23	0.000	0.692	23	0.000
2.25 HP/c	0.263	23	0.000	0.764	23	0.000
3HP/c	0.229	23	0.003	0.742	23	0.000
TOTAL	0.243	23	0.001	0.744	23	0.000
Total profit	0.233	23	0.003	0.735	22	0.000

a Lilliefors significance correction.

**Fig. 9:**
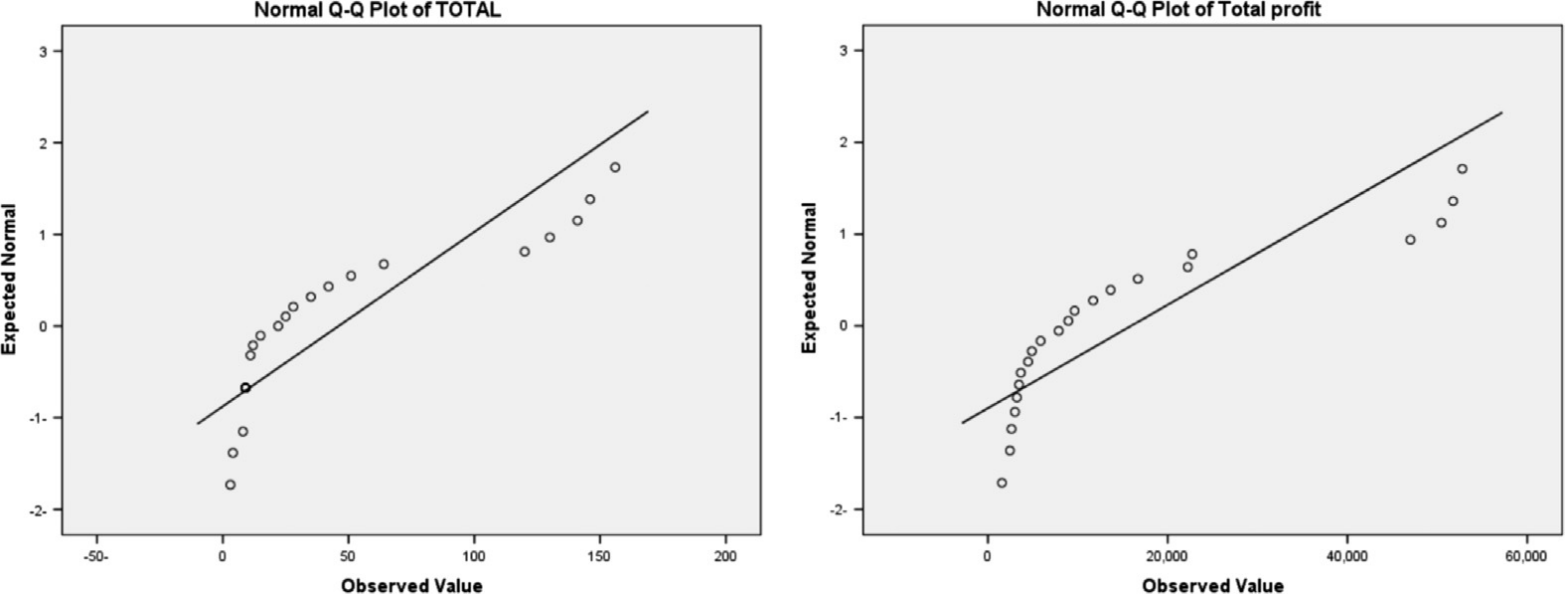
The QQ-plot of the total count, and total profit of all types of air conditioner.

The previous table indicate that all the types of air conditioner are not distributed normally, where the *p*-value is smaller than 0.05. The next figure indicate the QQ-plot of the total count of all types of air conditioner, and the QQ-plot of the total profit of all types of air conditioner (Fig. 9).

Therefore, we will accept the alternative hypothesis which said that the count of air conditioner don’t distributed normally. In order to examine the distribution of the data is the same or not for each classification (Sex, Cordon, Season), we will use Kruskal–Wallis test.

## 2 Experimental design, materials and methods

Kruskal–Wallis test has traditionally been used to investigate the same distribution of groups. In this research, a Kruskal–Wallis is applied. Kruskal–Wallis and other statistical tools have been applied to the analysis of economic data such as in econometric models. The null hypothesis refer to the distribution is the same across the classified variable versus the alternative hypothesis, which assumed that the distribution is not the same across the classified variable. However, SPSS version 24 was used for the Kruskal–Wallis.

Also, the level of significance used for all the analyses is 0.05. The result is displayed in Table 8, Table 9, Table 10.

**Table 8:** Kruskal–Wallis table to indicate the same distribution across sex.

**Hypothesis Test Summary**				
	**Null Hypothesis**	**Test**	**Sig.**	**Decision**
**1**	The distribution of 1.5 Hp/b is the same across categories of Sex	Independent-Samples Kruskal–Wallis Test	0.063	Retain the null hypothesis.
**2**	The distribution of 2.25 Hp/b is the same across categories of Sex	Independent-Samples Kruskal–Wallis Test	0.042	Reject the null hypothesis.
**3**	The distribution of 3 Hp/b is the same across categories of Sex	Independent-Samples Kruskal–Wallis Test	0.079	Retain the null hypothesis.
**4**	The distribution of 1.5 Hp/c is the same across categories of Sex	Independent-Samples Kruskal–Wallis Test	0.007	Reject the null hypothesis.
**5**	The distribution of 2.25 Hp/c is the same across categories of Sex	Independent-Samples Kruskal–Wallis Test	0.105	Retain the null hypothesis.
**6**	The distribution of 3 Hp/c is the same across categories of Sex	Independent-Samples Kruskal–Wallis Test	0.030	Reject the null hypothesis.
**7**	The distribution of Total is the same across categories of Sex	Independent-Samples Kruskal–Wallis Test	0.049	Reject the null hypothesis.

Asymptotic significances are displayed. The significance level is 0.05.

**Table 9:** Mann–Whitney U table to indicate the same distribution across cordon.

**Hypothesis Test Summary**				
	**Null Hypothesis**	**Test**	**Sig.**	**Decision**
**1**	The distribution of 1.5 Hp/b is the same across categories of Cordon	Independent-Samples Mann–Whitney U Test	0.487^a^	Retain the null hypothesis.
**2**	The distribution of 2.25 Hp/b is the same across categories of Cordon	Independent-Samples Mann–Whitney U Test	0.037^a^	Reject the null hypothesis.
**3**	The distribution of 3 Hp/b is the same across categories of Cordon	Independent-Samples Mann–Whitney U Test	0.104^a^	Retain the null hypothesis.
**4**	The distribution of 1.5 Hp/c is the same across categories of Cordon	Independent-Samples Mann–Whitney U Test	0.525^a^	Retain the null hypothesis.
**5**	The distribution of 2.25 Hp/c is the same across categories of Cordon	Independent-Samples Mann–Whitney U Test	0.379^a^	Retain the null hypothesis.
**6**	The distribution of 3 Hp/c is the same across categories of Cordon	Independent-Samples Mann–Whitney U Test	0.260^a^	Retain the null hypothesis.
**7**	The distribution of Total is the same across categories of Cordon	Independent-Samples Mann–Whitney U Test	0.211^1^	Retain the null hypothesis.

Asymptotic significances are displayed. The significance level is 0.05.

a Exact Significance is displayed for the test.

**Table 10:** Kruskal–Wallis table to indicate the same distribution across Season.

**Hypothesis Test Summary**				
	**Null Hypothesis**	**Test**	**Sig.**	**Decision**
**1**	The distribution of 1.5 Hp/b is the same across categories of Season	Independent-Samples Kruskal–Wallis Test	0.384	Retain the null hypothesis.
**2**	The distribution of 2.25 Hp/b is the same across categories of Season	Independent-Samples Kruskal–Wallis Test	0.577	Retain the null hypothesis.
**3**	The distribution of 3 Hp/b is the same across categories of Season	Independent-Samples Kruskal–Wallis Test	0.480	Retain the null hypothesis.
**4**	The distribution of 1.5 Hp/c is the same across categories of Season	Independent-Samples Kruskal–Wallis Test	0.676	Retain the null hypothesis.
**5**	The distribution of 2.25 Hp/c is the same across categories of Season	Independent-Samples Kruskal–Wallis Test	0.143	Retain the null hypothesis.
**6**	The distribution of 3 Hp/c is the same across categories of Season	Independent-Samples Kruskal–Wallis Test	0.082	Retain the null hypothesis.
**7**	The distribution of Total is the same across categories of Season	Independent-Samples Kruskal–Wallis Test	0.214	Retain the null hypothesis.

Asymptotic significances are displayed. The significance level is 0.05.
