# Supplementary material for: Statistical analysis of the count and profitability of air conditioners
Source: Data Brief. 2018 May 15;19:413–23. doi: 10.1016/j.dib.2018.05.035 (PMC5997838; doi:10.1016/j.dib.2018.05.035)
Supplement: Supplementary file 1 — Supplementary material [file mmc2.doc]

EXAMINE VARIABLES=VAR00004 VAR00005 VAR00006 VAR00007 VAR00008 VAR00009
  /PLOT HISTOGRAM
  /STATISTICS NONE
  /CINTERVAL 95
  /MISSING LISTWISE
  /NOTOTAL.


Explore


Notes	
Output Created	02-MAY-2018 17:45:47	
Comments		
Input	Data	E:\الرسالة2\2.publication\paper5\رسم وزوائد\pure_tech.sav	
	Active Dataset	DataSet1	
	Filter	<none>	
	Weight	<none>	
	Split File	<none>	
	N of Rows in Working Data File	23	
Missing Value Handling	Definition of Missing	User-defined missing values for dependent variables are treated as missing.	
	Cases Used	Statistics are based on cases with no missing values for any dependent variable or factor used.	
Syntax	EXAMINE VARIABLES=VAR00004 VAR00005 VAR00006 VAR00007 VAR00008 VAR00009
  /PLOT HISTOGRAM
  /STATISTICS NONE
  /CINTERVAL 95
  /MISSING LISTWISE
  /NOTOTAL.	
Resources	Processor Time	00:00:02.73	
	Elapsed Time	00:00:03.07	


Case Processing Summary	
	Cases	
	Valid	Missing	Total	
	N	Percent	N	Percent	N	Percent	
count of sales units for 1.5 HP/b	23	100.0%	0	0.0%	23	100.0%	
 count of sales units for 2.25 HP/b	23	100.0%	0	0.0%	23	100.0%	
 count of sales units for 3Hp/b	23	100.0%	0	0.0%	23	100.0%	
 count of sales units for 1.5 Hp/c	23	100.0%	0	0.0%	23	100.0%	
 count of sales units for 2.25 HP/c	23	100.0%	0	0.0%	23	100.0%	
count of sales units for 3HP/c	23	100.0%	0	0.0%	23	100.0%	


count of sales units for 1.5 HP/b


³/é<StwwoÜ¸ÑM²²ºYãÙY§:«xQ¬¬N?[½÷î®]»ÊËËS©TEEÅë¯¿>::9OFræ7onÝºµô¾ÖÖÖþþþìUp¡¾¾¾¤¤äå_È]caÙ²eëÖ­K¾õÖ[UUU±Þ¸´¸Øøì3wtt466Æ©ë×¯¼téRCCCæ¬ÂÕ-[¶L)èk¯½6mVïÜ¹ÝÍ^XYY)öIµµµ¹k,..Ã¤©SV±aÃì3g[³fM¤7s4²íYÅËêLr³¤îöíÛ1>þ|ËÊÊ¦-ôîÝ»ãèæÍïÞ8S#q4¦°###ÃÃÃZg_Ô&''9n:%±Æßºu+ÆÎì3ïÙ³gbbâòåËÉÑRÇÑïfUx²²ÚÔÔãäÙ³gïÝ»7ÓÄ7æ¦q4æ¬ÉÑ(q]¾|yr´¼¼<f:eïMGV£µ«V­Jæ²Ùg<gqTÙ+¾ «°ØYyöøÆIYQÇsçÎMQ©TjÊ%ÇÑX8Ózs×QÌÓÐ)O)ÏôFåÙ²OPV_|ñÅÁ'rÓéô´gK*8e¶ç>[Í^c,yã7:::ed!«Ék«Ék1sqIIIöI£÷ÅÑ¶¶¶äµÕáûïÙ³'9skkkÝ¶mÛ½÷6mÚ4V¹ïµk×bûþûïË*È*BV#SÝ¹sgrRò®¢°ûö8ÚßßLI³ß	ü×¿þ53ßÍ>iÝºu³g5ÓÝÄòåËã0y_±¬¬ÂRÍjÌD÷ïß_UU´mïÞ½cccÉIgÏ²Æ´2óIÓë×¯oÝºµä¾NyÒ¹sçjjjb'%ÏëNysoöcFgËTÇJ£ÙS>Û#« «ðôJ1¾|ùr'&&N8GëëëÝ2 «À¼%¯­Nqøða·È*0o£££ûöí[±bEòqÞ|óM7È*È* « «SP(2HFV¿ñ#øÌ3Ïü`é«¬¬L>+¬~Yî¹çþÀÒ·~ýzYUYUd@VUYdYUYUYUd@VUYdYUYEVdYU@VUYUYUYEVdYU@VUYUd@V)Ä¬UWWgvuu555¥R©ÆÆÆYdY«øaª¯¯/*úûÚkkk¯öööºººô÷÷w?èO>U@VÕ©6mÚtãÆì¬f+--Ã;våé¬ßF@VÕéÖ=]VcVºk×®<èÚµkf«¬"«sÍêÈÈHkkëèè¨×VYEV)«1mkkôN`à©ÍêêÕ«O:9ÚÞÞKëCqggg*jhhrzzzÒétrÒÅ§]ÂÕ¸_f9¿¬Õx`NLLÄ8ëêêfzÊB©¨¨¸ûvâ0û¡µµ5iü±cÇ¶mÛ6íÐ¬ÆýÖ$YÚ¬¾ýöÛçÎqîÛ·/yHÜ°aCL#ºýýýÉÄqÕªU±¤²²2&µÉ÷>|xåÊ±ðÓO?ãCqMMÍÀÀ@òa§wrr2ãããIqsðeu¾dx²zëÖ­äqØÕÕduëÖ­1¸téRô5õõõ1ÁÍ7-[|ï;ï¼Ù¦FY§LSÈsiii,Ãîîîì²/$ç.AVè¬Æá-[â°±±1³¤¤¤$û£±äîÝ»17Ý¾f7qL%gú¨Å´búÛ××ôuÍ5Ù'gÆq¦]¬<éY÷ÝwO>½sçÎì¬e3xæÌÎÎÎ;wîd²:ÓBg­Î2­¬¬Oòñ´KU'=«­äÖdÉ+¯¼rèÐ¡DJ×®]öæÍ£££»vízhVgyîííÁµk×¦¼ë8¦Â'OA¶¶¶N»YxÒ³6nÜ¼8Ú¨i¤´±±ñÆ±äøñãeeeË-ï½÷%«Ñæ¨iòf¨gªªªââât:ÝÕÕ5ídàÉÍ*²*«²¬È*²ê·(à¬^¿~ãÆ%%%¥¥¥ÍÍÍÉG_ZZZfy6wyæs8sß)D¦üÛìi$ÆÆÆÚÚÚb£V®ÙÙ9ßoUYÈ3«uuu§O¼ïã?ÍTÐY²¢©ÑæÇTÖÜ»$ã­·Þ:|øplQ45ÙÓ¼¾]Ve Ï¬.[¶,óvÜDfG1¾sçÎ5kÊËË/ðÐ¬&eM>»ïÃU«V%+ºuëVUUUf?sûo³gùGÚÉ´;¿oUYÈ3«gÏ­¬¬|ùåß~ûí+W®L)eKKKLûbpèÐ¡¹d5s4wßï¼óÎ#G¾¼¿ëÌþ|òLkÏ]8íÙ"ç±:ÇTõêÕ«óývYU<³bâA=zôhLLMÌiåÄÄÄ¼²»ïÃdWQÙ)SÉy$dn],..>qâDòÊqÌeUV/«ãããåååS²ì#"N×À¹û>LÚÕÕ5Ëÿs]¨Ùjön³÷(«²ðx³ºråÊd'Já/¾Hæv1ÛK¢ØÚÚzðàÁ[·nÅÙÕÒÒÒä¹ßÜ~y?MqæcÇå¹uqÏ=~øaz3kUYxìY½~ýúæÍ-[³ºæææ(h,|ùå388ßXWWÊh¦½êgÀ&wßaxx8Ü½7ïª=´ÉÑXQKKKø+!³vYUEYUYUYUd À²ZTTtúôé<^kìêêjjjJ¥R===ÉûÿøÖÐÐ á²*«ÀSÕèâèèè|³Z[[ì>¢½½½®®îËûû6:ölbyEEàÉª¬OcVO<¹ÿþGygliiiöÑ.DªOVex³qêõë×¿ùÃ33í!twwïÚµ+OLLÇ9c+x²*«ÀSÕhj!ÙêÈÈHkkkæ9äDGGGö~UY®¬ýû÷<yr^³Õ¶¶¶ÁÁÁÜÍÞe ²*«ÀSÕq655Í¶ÚÙÙÙÜÜ<44ý&¦ÞÞÞäMÂqàÉêgõÿ¹-~@_|1½8_±®_ÿú×;ü²N>=÷¬VWWOÈöôô444Ä<uÃÓNaÕGÊê¶m­þeóÅO/Î×÷Ö6üò¿ôØÌ+«ÈêÉê?ýÓ=üÓÿûøâ|mmùYdUVeUVYEVeUdUVYEVeUVYEVeUdUVYEVe@VeUVeUdUVYEVeUdUVeUdUVYEVeUdUVdUVeUVYEVeUdUVYEVeUVYEVeUdUVYEVe@VeUVeUdUVYEVeUdUVeUdUVYEVeUdõïª««3GzzÒét*jhh¸xñ¢¬²¬ÎUü0Õ××ýí­­­§NÁ±cÇ¶mÛ&«¬"«sµiÓ¦7ndgµ¢¢brr2ãããÉ,vïÞ½/<èùçé¬¬²¬N·î¬¬F/§?ûì³£:tèPII¬²¬>$«ÅÅÅq´ÓÀ¬"«ùgµ²²r||<y8Æ²È*²V·oß~òäÉÄakk«¬²¬æÕøñªªª*..N§Ó]]]²È*²úxÉ* «Èª¬Èª¬Êª¬²¬Ê* «Èª¬²¬Êª¬²¬Ê* «Èª¬²¬Ê*¬Êª¬Ê* «Èª¬²¬Ê* «Èª¬Ê* «Èª¬²¬Ê* «Èª¬Èª¬Êª¬²¬Ê* «Èª¬²¬Êª¬²*«²*«¬"«²È*²*«²*«²*«¬"«²È*²*«¬"«²*«¬Êª¬Ê* «Èª¬²¬Ê* «²*«²*«¬"«²È*²*«¬"«²*«¬Êª¬Ê* «Èª¬²¬Ê* «Èª¬Ê* «Èª¬²¬Ê* «Èª¬Ê* «²*«²È*²*«¬"«²È*²*«²È*²*«¬"«²È*²*«²*«²*«¬"«²È*²*«¬"«²*«¬"«²È*²*«¬"«² «²*«²È*K3«õõõ©Tª¡¡a¦5YdYÛ·oÇ «««eUd55551ÃÇà£>ú×íÞ½ûÙgU@VÕèéé)---**ÃîîîXrèÐ¡ï=è;ßù¬²¬>ÜêÕ«ûúú¾®Y³ÆÀ¬"«ùK¥RÓeUduÞbÚÛÛk×®ÅÌUVYEVówóæÍ¨iÌSã0Æ²È*²úxÉ* «Èª¬Èª¬Êª¬²¬Ê* «Èª¬²¬Êª¬²¬Ê* «Èª¬²¬Ê*¬Êª¬Ê* «Èª¬²¬Ê* «<Y]¾|ùºuë®_¿>66&«²È*Õ¢,UUU¯¾újÿää¤¬Ê* «²:o###===eeeÙ­©©iooUYdUVótíÚµíÛ·gÇ5Êª¬²*«s5<<ÜÑÑÑÐÐPZZÔ+W¦ÓéÂÊª¬²*«ù¿¶zóæÍ8irr2Z+«²Èª¬ÎUEEÅ¦M¼XVYe_[UYdGÍêÁcÎËÊÊ~øÃÊª¬²*«ùØ¼ysQQQqqqöK­­­­²*«¬Êê¼¥R©èèÀÀ@rôÖ­[q4Êª¬²*«óì"ócqéÒ¥8ú8>W#«¬RøY=qâDQãÇËª¬²*«ù8þ|uuuÉétúÂÞ²$«¬ÊêÒ «¬"«² «²ú0ýýýétº¸¸8ûµÕÌçmdUVYÕy¨ªªÊ®iÂldUYÍë;ïëêêò$°¬²Ê£fµ¢¢"²êµUYdÈêÅ#«ûöí»wï¬Ê* «|¹PÿoÕ[dU)«ÅÓñ%YdUVYdYUYÕ9èíí­««+++K^R]µj¬Ê* «²)ïTAKK¬Ê* «²:oÉ^úúú²³êÿ­Ê* «²×wÞ"«>`#«¬Êj/_ì¼0©i2¨¬¬UYdUVç­½½=wwgÎUYdUVóqåÊ+V¤R©t:þüyïU@VeuiU@VUYUYÛ;íj_VYe²Ù½~vVíj_VYÕG566öÆoDVÏ=+«²Èª¬>ªdw>·*«¬Êê45~>¼¶*«¬ÊêB¾eiÅ²*«¬ÊjþoYÊ¦Ëª¬²*«K¬²¬Ê*¬Êj^¯­>ÖýBÈ* «òk«ÓÖ4ñ8ö!«¬R°Y=räHtôÍ7ß½÷î;âè|ß¥µµµ¬²³³SVYåéÊj$0::åiáÒÒÒü.í­·Þ:|øðääd4µ¦¦&ô÷÷ÿÛ. «¬RÈYýõ×GFF8ð(YmhhèëëË^L§H¥R²È*Õ£GæfïØ±cù]ZôòÝwß*ÇTõêÕ«±$j=ð k×®­²Jaf59s¦ºº:3×t:ýñÇ?Ê N8ë×¯¯ZµÊk«¬òÔeueï£¦wË* «ÈêìÙ³çÃ?AooïÚµkeUº¬FëêêÊÊÊ?Ìôäí·´´Ä<5.äÆ²È*OWV;::¦ìS)ÆÇw]eU6«UUUÑÑ¾¾¾ì¬ÆÌUVeUYÿwÞ"«þ¹¬²*«yZ¾|yt´««+©i2È~C¯¬Ê* «²:Wííí¹»8sæ¬Ê* «²+W®¬X±¢¤¤$J¥ÓéóçÏ?Öë*«¬RÈY]d²È*ÕêêêÚÚÚ>c*«²Èª¬ÎCòoÌÍVeU «'O¬îÛ·odddrrRVeUõs«SøÜª¬²*«y>	k¦>#«²*«Þ	,«¬"«²È*K"«ûTYd§4«XYdYUYUYU@VUYd¥ÕYØ¬²*«óP<+»U@VeuiU@VUYUYUYdYU@VUYdYÕ¾¾ýí^|1½8_±®ÎÎNU «Èjfµ&½ç_6_üäðâ|ÕÔ¼ðÉ'x¨YEV3«µÿñÅKkddYUYYEVeUVYEVeUdUVedYUYdUVeUVYEVeUdUVedYUYdYU@VUYUUdUVeUYUYdYU@VUYUUdUVeUdUVYEVeUVAVUYU@VeUVeUdUVYEVeUVAVUYU@VUYdYUYYEVeUVYUYU@VYzYíèè(**UYYEVÕÄÄDSS¬Ê*È*²º:täÈLV÷îÝûÂþùT*%«²²¬>ÄíÛ·W­Z599ÉêgvôAÑÝYUUdõ!ZZZ.]ºôÿ®'edYÔëñ YUUduaúj¶*« «Èª¬Ê* «²ºtÈª¬¬"«²*«¬Êª¬Ê* «Èª¬²¬Êª¬¬"«²*«¬"«²È*²*«²²¬Êª¬²*«²*«¬"«²È*²*«²²¬Êª¬²¬Ê* «Èª¬Ê*È*²*«²Èª¬Êª¬²¬Ê* «Èª¬Ê*È*²*«²Èª¬Êª¬²¬Êª¬¬"«²*«¬Êª¬Ê* «Èª¬²¬Êª¬¬"«²*«¬Êª¬Ê* «Èª¬Ê*È*²*«²²*«²*«²È*²*«¬"«²*« «Èª¬Ê* «²*«²È*²*«²²¬Êª¬¬"«²*«¬"«²È*²*«²²¬Êª¬²*«²*«¬"«²*« «Èª¬Ê*È*²*«²È*²*«¬"«²*« «Èª¬Ê* «²*«²È*²*«¬"«²*« «Èª¬Ê* «Èª¬²¬Êª¬¬"«²*«¬Êª¬Ê* «,Í¬vuu555¥R©ÆÆÆYUUd5µµµW®A]]¬Ê*È*²º0JKKãð£>ú×íÞ½ûÙgÕ'!«¿ûÝï~¹~ô£=zt1×ø¿üÅã>²J!dµ»»×®]18tèÐº÷»ßÕ'$«/¾X]óßÚòóUTT´ö¥ÿ¼«ûãÿèqYeÉgudd¤µµuttÔÀOxV×­éÔ¯ö-ÚÖó¥?9¼h««~á[²¬²ä³:00ÐÖÖ688èÀ²*«È*²úH:;;f9¬Êª¬"«ÈêTWWeUYUdY¼dUVeYEVeUVedUVeUVedYUYUdYUYUdYUYUUdUVeUVUdUVeUVUdUVeUVAVeUVeUVAVUYUYEVUYUYEVUYUYYEVeUVeYEVeUVeYEVeUVedUVeUVedYUYUdYUYUdYUYUUYUYUYEVUYUYEVUYUYYUYUYYEVeUVeYEVeUVeYEVeõ+ÊjãùöÏþóOKSS¬.O]¡êÏþó"ßþ¹¬"«Õ²o<WþÍoÄuq¾ÊË¿!«¥¨¨hÑî¸øÕjV£sycþ»ò²_¬Udµ0³Zõ­¿[äÕ|VmÓâ«°³µ[´[2~Ö­IVUYUYUYEVeUVeUVeYUYUYUYUYUYUYUdUVeUVeUVUYUYUYEVeUVeUVeUVUYUYUYEVeUVeUVeYUYUYUYUYUYUYUdUVeUVeUVUYUYUYEVeUVeUVeUVUYUYUYEVeUVeUVeYUYUYUYUYUYUYUdUVeUVeUVUYUYUYEVeUVeUVeUVUYUYUYEVeUVeUVeYUYUYUYUYUYUYUdUVeUVeUVUYUYUYEVeUVeUVeUVeõ	ÐÓÓN§S©TCCÃÅeUVeUVeYÍ_kkë©S§bpìØ±mÛ¶Å ¿¿ÿßtáÂ;ùúÇÜzð¿oÿ_WþÇâ|»æùÅ·*¿ì¿êêÊÊû¿9¸h«K?_ÙÝÝg±DçmÓâ+Vw§@ýæ7¿ùÎêÿ´h·dü¼ôÒwò¾¶ßûÞ÷dUV£ÉÉÉWWWÇ`ÇE9yæT¾¾þõ¯ÁÜ¤Qao]~óøeoYä[òk_ûZ~EqUî¹Ï?ÿdõqµ)ãáááûÛßù]¼x1~yFFFòîÿý÷ãBýáþñ¼víÚBÝºïÿû?øÁuë~òêÖEPÛÛÛåGV¯øã43.))yrn YUYUYeéeµ²²r||üËûOÇXVeUVeUVÕümß¾ýäÉ1ÃÖÖVYUYUYEVówùòåªªªââât:ÝÕÕ%«²*«²*«ÈjUYUYUduÁüéOúÙÏ~¼î[xþð?=z´Pï»ßÿþ÷|ðA¡nÝoûÛóçÏêÖýêW¿ºråJ¡nÝ/~ñ¾¾>®²È*ÈêW+wÅsÙkñfhh(ÙeÕLµt··«««©©)®^cccBÚºÞÞÞÕ«W'×-Ù¹]!Ýq¢¢¢Âû±ÌÞSAÞwÈjr÷Q»ä	Èõõõ¯Ù7aÉmommmòÚ[]]]!m]<¤=6±ÉÊéñ'Qæ'³¶îÌ3mmm³oB!=È «ó»âÜ%O¸M6Ý¸q#;«³lÂÞÞÒÒÒÜº.Äð6íÐ¡CGÉüdÒÖES?þY·Üç.YwpVVgÙ¥»½ÝÝÝ»ví*°­ùÜwÉÇ0iÓnß¾½jÕªGæ'³¶®¶¶¶¹¹9®Xlãõë×õYÍGî>Ø½Ï=«³lÂÝÞÖÖÖÑÑÑÜºdG´i---.]ÊþÉ,¼;.$Ï4äÖ!«ùÈÝGñ»×â¹guMXÛ¹uÙSBÚ´)ÿX­Pï¸LuëÕyËÝGñ»×â¹guMXrÛÛÙÙÙÜÜ<44Tx[W[[ÛÛÛûåýw;Ç6ØûYH[÷]ò7_ßwÈj>¿í¹û(~b÷Z<÷¬N»	Kt«««§LzfëzzzbºaÃd.^HwOf!m]wwwÜwë×¯²ê¬¬¬¬²²²È*È*È*È* « « «¬òºtéÒ5kR©TiiéÆé§öÿÿ3¸¯ì×æÑ®ÀÞatt´¢¢bÚë366Vc½0ûÛKJJªªª99çððp,_¾|ùp×¬²ôtwwçt:yhÞ¾Ád5[conÏjÆ»ï¾záÂ8ºuëVYYeñª|×Ý»wc<>>¾¤³úWf¡n«W¯®Zµj^<y2·µµå±	¹YÍüí·ß£ÕÕÕÉ£GÆÑýû÷Ë*Èj¡9uêTÌJJJvîÜåüqCCC*ª««;wîÜL§¹¤1illozuoooîÄ%÷:L»®Ù¿ëúõëë×¯Ï<)zçÎdùíÛ·ãh,L®@Ì§½Ú~øammm'®j^ì,EË-¶ÜízèJ§½)f¿>øàòòòuëÖMQ±ù3Ý;vìHú<sûÞï=zV3KâÚ&ãW_5Æ&dÎ?U÷?~Üo%²ÊRkÙ×?ýéOå~úéòK.Í1*Ù¢+äLëý»¢(Ù§®Y³&YkÌ^ÉÉ½¹Ïsfê;ÓÅÎ+«¹·ÀìYèJs¿å¡wP"y5[×,sÄèúK>sæÌL7Â´¦­>|8ÖÔÔ$KâÏ8Ú××7íEµ··ûÝDVYÖ®]Ì®nÜ¸ý¨<¶îÞ½;ã0Æ6lcT^íµÉÉÉ?%ïUer3ßue+..¦M&nÝº5ÓËq)Æ/_ñ¶mÛæx±rL¹<VúÐmß¾É0ÇÌØ²eË²eËâG"6áÐ¡CÙäÕ)='Å%Ç&ÇÌ5sîÝ»7Æqã¦¦&¿È*KR<´MûØ1åÉkxqãÒÒÒ9Fettô¡!Éo]ÙvîÜ'Å£óºuëb=<<9é/¾8pà@æÄÜJÖ­¼¼ü¡;÷¬>ôr	y¬ô¡7Úìþrn¯hFö²¹è·ÏòNàººº÷ß?9éêÕ«±0î ìsÆx```¦5¬²d²LòH]rÒLµx¬YYZhóæÍÉ[dåñØG<Ì¿§½ÉVg¤Í~±3Å#¿[`ÊÙæ»Ò¼o´¹dõW^?2´ÇË¾ä<²:íªO>'íØ±#ûÉëú×Ö²Ê<	|þüù¾¾¾,[¶,Y<Mýcæ!I>ýôÓñk¯½6¯¨efr³¬k.¸÷nöü&éÍíÛ·/^¼8ÓuK^íèèûÅæþ-ß-0û­1ËJ§#Þh³mÓ¦M±üõ×_qüuã-[¶,lVãÂã¤Ì¡sÆºb¼oß¾ç¾Ód¥aÊ[Ó¼.íÊ+ÉIë×¯Ë»T¦]¾|ùL¹e]³<4'ddW®YXVV###S.êÌ3SÖ-ýb³åwÌrkÌe¥S.9¿mÌË2¿víÚÂf5:ýgMîyöìY¿È*KÕñãÇ+++ØÜ»w/»¸É§Dêëë³'v7nÜÙ^<ÚÖÕÕeªüÐ¨$K¶3­kæÁÁÁÖÖÖä4Ådywwwò±W^y¥¿¿¦GÆõuÅVÄ=f3gºØlùÝ³ÜsYiîMÇ6¬¨Zmmml`SSSLúç>ÙcV?w2?lÙ°»ÃlU@V@V@VYYYYdddUUUU@V@V`éû¿Ìµ?5qQIEND®B`


 count of sales units for 2.25 HP/b


áÂ^¿~ýH¿òÊ+qvïÞ½÷q6f&q6²÷ïßKyUÇJFº1'ÖÓ·nÝé°3sáÃONNvww'gc`gcÔ¹¤$²J+NÖúúúÞ¼ysÙÙÙùàÁÙ¿1B³1rMÎÆq¶¤¤$9»aÃ8;::yé´5¦¿7ibb"dnmæÂ!tæÙ»wïÆtÀìÕ_¬Ò2È~æôàà`kRyþüù¯*JM»æ83g[oöÃÅô¥1öÜòlo]ÎVY¥%kÒ_~yüøñäÝK 6fó³f®±¦¦&æ¼ñÆ]]]cccdÈ*­Y×Y×;cüÓ?*Î¶¶¶&¯³=*aøðáÃÉÂÍÍÍqöÀ<¸÷îîÝ»sËoÞ¼Ù?ü¬Y¥U"kX8í)Ù&%o2ZZZâìððp20Í|oð×_õf^´mÛ¶Ü²¦éM*))ÓäÆdÈ*=Ã²ÆxôèÑ£ååå	o¯¿þúÃ:;;×?:00°ÿþÂGÅ uÚ;Î?¿yóæéÆEÉ¼ÓÞî¹pkc±¸Ð:VlOûÀY%²Jkºä©ãîîîüè£âlMM-#UR>%¯³NëäÉ¶DVIù4>>~äÈ7&ÏÇÄo¾i³Hd$¬$¬$uÖþò¿´KÒ*Bd]æîÞ½[PPÐ"I«"&ëõÛßþö%iUDV²UÈ*²JYEV²J"«ÈJVI"«È*IdYý6J"«ÈJVI"+YÉ*IdY%¬"+Y%Ud%«$Ud$²¬dDV¬DV²UÈ*²JYEÖÿÏdeeeúloooEEE*ª­­½|ù2Y%Ud]@ÝÝÝ555UssóÇgÎ9pà@Lüñx¼/¾ø¢°°Ðo£$²¬ÓÛ½÷àà`æ^UZZ:55ÉX¶µµõ¹¬þù¼wâÿüçÏ-_÷îñ8"¬dÂëÎØ«R©Ô´é%³¬ÿ×Þþßú_'ß~ùÇ?þ#ÈJÖ§'kAAAz:ø|¯³UYµd-++Hi²JZ³²nÙ²%yßIRÌy¢Æ±ä=¤ÝÝÝ¹ßpa­ Y[ZZÚÚÚb"NÉ*iÍÊó®ÉÉÉÓêêê'=º#;;;câÚµk¥¥¥¹ßpca­,YãW^^^PPPQQÑÓÓCVIkYÖwÞyçüùó1§GIÝ±cGÃÝáááo^±¡¡!æÅÐ6ùÞ'OnÚ´)f^ºti¡Ë/^¬««ËýÓkùe]hd´Fd½uëÖ¡Cb:Nc°,¹ÿþ®®®¸zõj1é*..N¾÷½÷ÞV3ßÙ´ÆàxÃ1?:Ç@hÎEV²JZq²Æé¾ûâ4&sÓ.&jÞ»w/F¨---é'lã4ùc6ó)äñ.3^Õl¬d´eÿý÷Ï;wðàÁLY>|¹äÖ­[;::®rçÎ´¬³qÌ:ãg sË:ÛÂ"+Y%­DYGFFbD¼ÚÌÙ³gÏ'b"4ñÅkÆÇÇ:4§¬9ªªªêëëÆÆÆÜ²Î¹°ÈJVI+QÖhçÎÉ;ÓÖ¨¡i]]Ýàà`Ì9öìúõë?øàÅÈÚÛÛ[[[Ð;vfæô¬d´reYÉJVIdYÉ*¬"+Y%­YvîÜYXXXTTÔØØØßß3æÿÝÌçäq¼ÕÕÕ3â.444Ä]Ø¿ÿØØØsr|;YÉJVIK)kuuõ¹sç¦õùçoÚ´i6Dç|T°<?9ëëë³oÆ-[£éÅqNo'+YÉ*i)e-..Ê>FDLß¹sgëÖ­6l¸xñâ²&¸&:Í>8b"Ýºu«¼¼<ùwâÄS§NeßÌÏ¹&ÊãÛÉJV²JZJY;;;ËÊÊvíÚõÎ;ïvmMMM'OLX¬é³ÙG|ï½÷¶oâØ±c¹XÎ>¾ÄíÛ·Ãæð8ûfÔÕÕ%yýäOS³çäøv²¬RÖ(¼	SO>ÃÓä?Ê¥ñk2¸¬ÙGë6y5wAñÉQ³oÆ7ª««Bî8qNo'+YÉ*ieM711±aÃi²&ôlpöÁSzzrüÿ×cÖù.qhh¨¦¦fÆ9ó<Ú"YÉJVIuÓ¦MÉ!¢/¿ü²¡¡!&?~ëÖ­XlNY»ººb<	pÄoÅ)>sæÌ¢ ÉºUUU½½½1¶êÛo¿=ã9ßEV²UÒÒÈ:00°wïÞâââk666¢1s×®]1èLÞ´ûöêêê¾¾¾ô5Ì6ÌüÔMöÁ£±±±sïÞ½Å8Ä+W®lÜ¸1®ùÐ¡CÉ;YÉJVIËðl°ÈJV²J"+YÉJVIdYÉ*iµÊóÏ;ÇË===õõõ©Tª®®®··7æôõõmÙ²%æÔÖÖvww3¬d´FeÇÇÇ*kUUUrdöööêêêP;;;c"æ2¬d´Femkk;zôèbÞ:!ÝÅCkæ¬Ö¨¬qºûöofÿDM,~ýÐ¡CÉôäääbÉÈ2¬d´veV×<Æ¬÷ïßonnN?ÔÕÕUVVÆ<²UÒÚ5:zôh[[ÛÆ¬###­­­£££ÙWùßfDV²JZ²Æ¸sAÿ¾ôÊ+ñPù¦ä?ÌôôôÄEÌ#+Y%­iY£sçÎÍ_ÖÊÊÊiÃÙÞÞÞÚÚÚ­îØ±cÆ¬ÈJVI«_V¬dDV¬È*²UYEV²UYÉJV²J"«ÈJVIdYÉ*¬"+YÉ*¬"+Y%Ud%«$²¬d%«$²¬dDV¬È*²UÈJV²UYEV²J"«ÈJVIdYÉJVIdYÉ*¬"+Y%Ud%+Y%¬d%«$²¬dDV¬È*²¬È*²UYEV²J"«ÈJV²J"+YÉJVIdYÉ*¬"+Y%¬d%+Y%Ud%«$²¬dDV¬dDV¬È*²UYEV²UYÉJV²J"«ÈJVIdYÉ*¬"ëëÊ+555©Tª¶¶¶»»¬È*².ªÒÒÒÛ·oÇDVVVUYEÖEµyóæÓ_üâ/>Þ-[bPKVIdYç®···¨¨(ö³8½~ýzÌùì³Ïþ÷ã>|xÝºudDVuîb<Úßß»uëVÏK"«Èº¨R©ÔÓdDV5bÚ××7oÞñ+Y%Ud]TCCCÉâ4¦É*¬"ë¬È*²¬ÈJV²UYEV²J"«ÈJVIdYÉJVIdYÉ*¬"+Y%Ud%+Y%¬d%«$²¬dDV¬DV²¬È*²UYEV²J"«ÈJV²J"«ÈJVIdYÉ*¬"+YÉ*¬d%+Y%Ud%«$²¬dDV¬dDV¬È*²UYEV²UYÉJV²J"«ÈJVIdYÉ*Id%+YÉ*¬"+Y%Ud%«$²¬d%«$²¬OÿëÇÿð_âWè*þW¬÷ÿ±ÕCDVuUÉú?þûßÿÇ-ÿþòoO>ý¯¸Ë4È*²®6YôÃ­DV²¬È*²¬YEV²U"«ÈJV²J"«ÈJVIdYÉJV¬"+YÉ*¬d%+Y%Ud%+Y%²¬d%«DV²¬dDV¬dÈ*²¬YEV²UYEV²J"«ÈJV²JdYÉJVId%+YÉ*¬"+YÉ*Ud%+Y%²¬d%«$²¬dDV¬dÈ*²¬ÈJV²UYEV²U"«ÈJV²J"+Y§WRR²mÛ¶¬ÈªÅÊúååå/½ôÒðððÔÔYÉ*¬dÍ§û÷ï÷ööÖÕÕ­_¿>SÙÍ7···¬ÈJÖü»yófKKK¦¯q¬dDV². ±±±®®®ÚÚÚ¢¢¢LS7mÚTQQY²UYÉº¨×Y&''ã¢©©©à¬dDV². ÒÒÒÝ»wo0Y%UKÿ:ëâ[[[7mÚtåÊ²U"«Ö¢¬Çk2½~ýúüä'y_Õ[o½uòäÉ©©©`uóæÍ1§»»ûïý÷ß_·nYÉ*U«SÖ½÷ÆnQPPù²ksss~×V[[Ûßß9ç§?ýiñã÷»ßM¥Rd%«DV­NY¹Ø-FFF³·nÝ³13ïk!iQQQXoÜ¸áÙ`²JdÕ59@DwwwröêÕ«q6ïÛÄØ÷£>²U"«Ö¬ásY=6¿k+++Ë¿¬YµßÁtáÂÊÊÊÂGUTTx1ï«:|øð§~/¾ø"YÉ*UkQÖ%lll¬©©)F«d%«DVõGV²JdÕjuxx¸¢¢¢   óuÖôpÈJVId%ëÂ*//Ï5)ïOÝ¬YµÖeMLíééñl0Y%UK kiiéSÞ-ÈJV¬ZÍ²^¾|9v#G<xð¬dDV-Í³ÁÓò&²J"+Yó¬`¦¼¬ÈJÖg&²U"«ÈJV²J"+Yç]___uuõúõëWgû5d%«$²uîººº¦½q)&ÈJVId%k>%Ç`êïïÏ5ïÿÏJV²JdÕZ5­&!ëÔÔOÝUYÿÏµ¤¤$9ºaj2ùÌÉJVId%ëjooÏ>RDGGYÉ*¬dÍ³k×®mÜ¸±°°0JUTTpÁÉ*¬df"+Y%²¬d%«$²u!ïvD~²J"«FÖôQø3euD~²J"+Y ¾ñÆ±tvv¬ÈJÖ%(9RÏ³UYÉº4¬vww¬Èª%~ÓÆÉJVId%ë¢ÞÁ.X +Y%¬ÏFd%«DV¬dDV².âuÖ'zÈ²U"«Vùë¬3ô$AV²JdÕjõÔ©S±[¼ùæããã÷îÝùåãì'|âÙ`²J"+Yó©°°pÚngÈJVId%kþ²¾öÚkccc÷ïß?vìYÉ*¬Êÿçzúôéì×YÏ9CV²J"+Yó¬£££²²2JÅøµ¢¢âóÏ?¢·¬dÈªU.ëS¬dÈ*²¬ÈJÖy×××W]]½~ýúä¸d%«$²5Ïººº¦q)&ÈJVId%k>ÇnÑßß)k_ÉJVId%k^ßù¨d"dòÏÉ*¬ÊÿçZRR»EOOOj2QVVFV²J"+Yó©½½=ûHd%«$²5Ï®]»¶qãÆÂÂÂT*UQQqáÂï&«$²õ¬dÈªÕ,keeeUUÕàà YÉ*¬ZYÿ|nÌJVIdÕÒÈÚÖÖ»Å#Gîß¿?55EV²J"«¾YüçY§åó¬dDV²æÿlpv©T¬dDV²zo0Y%Ud%+Y%²jÈú¤_L%+Y%²jíÊú%+Y%²¬d%«$²¬dDV¬dÈªgUÖ9RY%¬« gAVId%ë3YÉ*Ud%+Y%¬d%«$²¬d%«DV¬dÈ*²¬È*²UYEV²U"«ÈJV²J"+YÉJVIdÕêµ««+Ç®FV²JdYÐäädzWûë_ÿ:õx_ý5YÉ*Udo'N8uêTzWkmmÍþ_:Ï?ÿ<YÉ*Ud»Û·o744ÄÀ4½«Ý¸q£óñ~ýë_¬YEÖ¹kjjºzõê7þÿ«×YÉ*Ud]ôíx<²U"«ÈºdÄ³U"«ÈJV²J"«V¤¬9"+Y%²¬d%«$²¬dDV¬dÈ*²¬YEV²UYEV²J"«ÈJV²JdYÉJVId%+YÉ*¬"+YÉ*Ud%+Y%¬d%«$²¬d%«DV¬dÈ*²¬È*²UYEV²U"«ÈJV²J"+YÉJVIdYÉJV¬"+YÉ*Ud%+Y%Ud%«$²¬d%«DV¬dDV²¬È*²¬YEV²UYÉJV²J"«ÈJV²JdYÉJV¬"+YÉ*¬"+Y%Ud%+Y%²¬d%«$²¬dDV¬dÈ*²¬YEV²UYEV²U"«ÈJV²JdYÉJVIdYÉ*¬"+YÉ*Ud%+Y%¬d%«$²¬d%«DV¬dÈ*²¬È*²UYEV²U"«ÈJV²J"+YÉJVIdYÉJV¬"+YÉ*¬d%+Y%Ud%+Y%²¬d%«DV¬dDV¬È*²¬YEV²UYÉJV²J"«ÈJV²JdYÉJV¬"+YÉ*¬"+Y%Ud%+Y%²¬­§§§¾¾>JÕÕÕõöö¬YEÖEUUUuíÚµhoo¯®®&+Y%²¬KVQQQ¶µµµ>^KKËºuëÈú¬ÈúÕW_ý|ùz÷Ýw=p¯þå_þe÷´ßþö·dÕJõúõë_ýêW?z¼]»v¥R)²>+²Æ#N<pÄ6ú_Mÿí?Çª³FúÑ~XQñ½eÙÓb7²jEËzÿþýæææññqÏ¯Yÿnkí²ÜëË¿=ùÂÈY#Åûù²ìiñBV­hYGFFZ[[GGG½7¬dYEÖÅvåÊÆÆÆ°3Ç2d%+YEVu¾UVV>YÉJVUdâ¬dYEV²¬"+YÉJV²Ud%«ÈJV²¬dYÉJV²¬"+YÉJV¬"+YÉ*²Ud%+YÉ*²¬d%+YEV²¬d%«ÈJV¬d%«È*²¬dYÉJV²¬"+YEV²¬d%«ÈJV²UdYÉJV²¬dYÉJV¬"+YÉJVUd%+YÉ*²¬d%+YEV²¬d%+YÉJV¬d%«È*²¬dYÉ*²¬"+YEV²¬"«ÈJV²Ud%+YÉJV²¬dYÉJV²¬"+YÉJV¬d%+YÉ*²Ud%+YÉJV²¬d%+YEV¬d%«ÈJV¬d¢mÜøÂ¶m·,_/¼P944´,÷ú¥Z~ðe¹×ßûÞ¿&«ÈJV²®fYãáòã>²,_±ê¯¾újYîu°¿_Ër¯ÿæoÉ*²¬«e¹×ñUù·ÿf¹d±c ·,÷ºêßý-YEV²¬d%+YÉJV²¬d%«ÈJV²¬dYÉJV²¬"+YÉJV²¬"+YÉJV²Ud%+YÉJV²¬d%+YÉJV²¬d%+YÉJV¬d%+YÉ*²¬d%+YEV²¬d%+YEV²¬d%«ÈJV²¬dYÉJV²¬d%+YÉJV²¬"+YÉJV²Ud%+YÉJV²¬d%+YÉJV²¬d%+YÉJV¬d%+YÉ*²¬d%+YÉ*²¬d%+YEV²¬d%«ÈJV²¬d%+YÉJV²¬dYÉJV²¬"+YÉJV²Ud%+YÉJV²Ud%+YÉJV²¬d%+YÉJV¬d%+YÉJV²¬d%+YÉ*²¬d%+YEÖYêíí­¨¨H¥Rµµµ/_&+YÉJV²¬ª¹¹ùã?3gÎ8p &~ÿûßñx/^nÝòíg?ûÙþ§ÿóÙ?=ý¯íÿÿPÿoeÕ[ÿkSÓ¾?,GöÙ¦Mß_ýÁéÃ%%ÅX¦âárYîu|;~Så^×××ýÓÿsYîõ÷¿_ûù²¬:Râe1»Èú+--ÊÊÊhmm.«ÔæÛóÏ?×ð¯£äÆ/×ª£eÜàIËÔ2ÞëXuüõ¹,÷ú[ßúÖÚüýý<ïöï|BdÓ¦ÿüç?ÿß¬<x°,7oxx8~~÷»ßÙcæßo~ó¢¢"ÛaA½òÊ+»ví²Tmmí[o½e;¬ÓqUz:þ[i¬d%+Y¥gLÖ²²²o=Ód%+YEVuQµ´´´µµÅD677¬dYEÖEÕÝÝ]^^^PPPQQÑÓÓCV²UdYWsd%+YÉ*u)ûÓþôî»ï~ýõ×6Åüëïïÿå/i;,¨K.öÙg¶Ãjkkûâ/lU$²J$².¦ì£ÏçøÆkªÑÑÑÌdÙz¹»÷nr±Ù6­ÙßDÖg¾ì£gÏYãutt´¶¶Úzó©»»»¦¦&ÓÄÖqÙßDÖg¾ì£gÏYãÅÃ§­7vïÞ=88éDbëÍ¸Åìo"ë3_öÑ³ç¬ñªªªcS444ØzsÿÊe8cØz3n1ûÈúÌ4ã~|ãeldd¤®®ÎÖ[96­7ã³¿¬Ï|ÙG3^áÇ7^Þ¦=xÙzs:cØz¹eµ¿¬ÏjÙG3^áÇ7~úUUU'cÆÆF[oANäØ ¶Þ[Ìþ&²>ó¿ÌÙG3^áÇ7~ú]¿~½¦¦&Jmß¾=ìl½91ã±õrl1ûÈ*IY%IY%I"«$Id$Id$¬$U$²Ú$U$²JDVIDVIÈ*IY¥Ü]½zuëÖ­©Tª¨¨hçÎÚSµ¿*»Kµ)Î;W]]×S^^þæoNMMÍs>Õ<ïfæÌÌo/,,U:u*½äØØXÌ/))Y	?2¬ZÑ]¿~=***ÒÂ---«FÖ<¶ÆlóçÏO£ñèÑ£ó««kÉeM÷þûï'^¼x1Îîß¿¬"«ôD`K¾ëÞ½1=11ñLËºÈ³$¢±±1®äÄ1ÝÙÙÓ6lç2mmm1ÝÚÚÇ]Ë5|çwâleee2çôéÓiËÉ*²êYíã?ñPaaáÁã.=ÿóÏ?¯­­M¥RÕÕÕ1í¡3ûA3uuuñ[¶léëëË¦dß×û»¶oß~vôÎ;ÉüÛ·oÇÙÜÎx³?ýôÓªªªX&njÜà9¯6óÙeß¯9W:ã¦ÈýúäOBÄmÛ¶e^OÌL~ÊSSS±X¬qÚºf[æå_NLÂýà/kzNÜdú¥^³q×ÒKÆþYþ¨³gÏúmYõaÙo¿ýv2ÿÒ¥KÓÍ¯^½:OW2Zæ4r¶uåþ®@%óÒ­[·&ócóCìÛýÄfàÙ®vA²foÜ²Î¹Òìoó<­:c.rìË¸ÓÖØÑÑ1ÛÆ±Ç¬'O³7oNæÄ_$q¶¿¿Æ«joo÷;+²j¥÷â/&c¬ÁÁÁÌ¸äaôW^Ç¾8é;vÌÓW_5Æ:ÉËu1¾É1Yèº2+((fS&Ýºuk¶×ÓSLwwwÇôæyµK²¦]C+s£9r$Ù3^[¤KJJb½aóYfß¾ÅÅÅ±«Ä];qâDæyÈ:­Ó§O'CäX]_÷L%½þúë1§1]__ïwVdÕJ/Åf¤+<ùÉëyqù´á®Ïç9À<ÖÙÁã¢x Þ¶m[µÇÆÆÒùåÇkhhí$kÌ,ýrc«¿¬sni×ÇJçÜh9íSZZäYâôS¸9ngîaÜêêê?ü0¹èÆ13~pKÆôÈÈÈlkÈª(köG/æ©]rÑl`<QYc¬íÝ»7yÓlccc2?¦ãìñãÇQø7 ¹×j¹¯v6'òÛÓ[èJóÞhÑÍ7c$|ôÑGZfÏ=ñ÷G¢~Üà_£ý&¯×YÓ;w..zùå3L^ñ_g[£DV­¬g/ÐßßÅÅÅÉüäùÒÌ'ÓIXºtéRxüê«¯.È¦Çsér¬k>ï½wï^æh&!çöíÛ/_í¶%¯ÅvuuÍÿj³ÿÉoäÞ9V:íóÛhwïÞ-//K3?B:ÏevïÞ3_íµ?ß¾K+k~oT²d¬+¦9ÓÓÞ%U+±iï`J7¿éC×®]K.Ú¾û|Þ2íl2ñ³9ÖãQ8ù ]ú1wÓ¦Méë×¯Óû÷ïO»ªik6r_mfùm[c>+vÍùm´£GÎyËg[&®Ú(?¶K+kPùOöÍèììô;+²êèìÙ³eeeÉ§n<xnòÑÌáÝàà`ùâµºº:ó®$kÙÖãQxtt´¹¹9ùtM¸822Ì¿~ýzòY=öÏöáÈ¸=±®¸1Lqgú¥ÍÙ®6³ü¶@­1fo<6Z2Í-ke¶ªªª¸ãõõõ/_ùa²&	¥wÂÌOÝÄÉ§nDVIDVIÈ*IY%IY%I"«$Id$¬$¬$U$²J$²JDVIÈ*IÈ*IY%I"«$Ik©ÿ¼`g:xÜàIEND®B`


 count of sales units for 3Hp/b


úôé.))é§z*N¶´´Ü¸[LÄÉ^ÆÉÈÝ¼y3vúE8p`jj*éVUUÅ¸Æé°3á½÷NNNöôô$'c`'cÔ¾¤$²Jy'ëbº¦¦&ììì¼uëÖlÃß¡ÆÉ¹&'ã8YVV,--£££éçN»ÆÔï&MLL¬ÁmSSS2¢M_8N?yýúõ½û+UúdÍ>?úòåË	®Iä©S§f¼¨ÂÂÂi'cæl×yábêÜNmy¶.g?)¬R~Étþüù&¯èVUUÍ¸Xá´1kÌû5ýëëëcÎsÏ=×ÝÝóæM²Jd¬Éû¬Éû1~é¢¢¢ô³Æï'ÛÛÛ÷YoÞ-axïÞ½ÉÂmmmqrçÎ·nÝº~ýúöíÛ³Ë/Ù7ß|¬Y¥e"kX8í%ÙÝ»w'g%2víÚ'iúgÿûß§F½égmÚ´)»¬)zÊÊÊâgòIc²Jd°¬1Ý¿eeeÂÛ³Ï>ûöíä¬ÎÎÎÀ5©o îØ±£èn1Hö¤S§NÕÔÔÄH7ÎJ^àöqßôcÅåÖq¥Áö´/üU"«´¢K^:îéééÉÉÉ·Þz+NÖ××Û3Y%-¤äÖi>|ØÈ*i!ïÛ·oÝºuÉkÅ1ñÂ/Ø-Y%I"«$I"«$IdýKúÓ:$iY!²þU»~ýzAAÁ.IZ¦ùÆ0Y?Y?ÿùÏÿQid%+Y%¬"«$Ud%«$²¬d$²¬DVUÈ*²UÈJV²JYEVI"«ÈJVIdYÉ*IdY%¬"«$Ud%«$¬d$²¬DVõÎÛ·o···­_¿þìÙ³dDVuQ:tèðáÃSSSÁjMMMÌùÝï~7xoôQÐë±'¬"ëýkhh¸téRúÂ®ÊhõêÕ¾Ëþô§?]÷ýð?ôä"UdÍA¯½öZqqqX?þøã1fYÿËÆK¿:«ÿø?þáßØÃÜÑú_É*UdÍMo½õVL MMMâV²J"«V¬éãW²J"kª~øwÞIìèè9ô9ùâÅqñlÜÐÐÐÓÓý¬ªªªdÎ3g8/²îÝ»÷ý÷ßOn³Gy¬ÈúoÏË«Vf1?ëêêôè6ìììÞÞÞòòòìgµµµ%ð=ztçÎÍYoÞ¼ÙÚÚò455]¾|¬È.ëK/½têÔ©ûöíKÝ²eK<s»CCCÉð1EcNEEEmß=|øðúõëcæ~8ß'ç®®®ÆÆÆìg¯SSS1111Q]]ÍÑ|u.UÒuxxxÏ=1?ûúú%wìØÑÝÝçÎbc¢¾¾>¦câÊ+k×®M~÷W^	ùÕiïµMûnÂ´+ÁqiiiÌOÎrVúÅÎövYÉJVIù%kü|ì±Çâg2LLæ¥»qãFPwíÚÄ&ËÄÏd@ú­yr§fÆ³R3c8JV²JZ²¾öÚk'NØ½wº¬·oßN_rãÆ'O<öìµk×R²N»9Yç2MÎ_'&&Wgc¬d%«¤¼udd$ÜJÞmMæ<úè£/¾øbL¦Ég?ÃÚ+W®ïÙ³ç¾²f©¶¶öâÅ1Ñ×××ÜÜý¬"?~<&âg[[GÉJVIKCÖhëÖ­É'SÖ¨¡icccòÙÏcÇ¬]»ö7ÞX¬1$Ý²eËèèhú¯gÕÓÓSYYYPPPUUÜr¬dï²¬d%«$²¬dDV¬V¬[·n-***..nnnNþg°ÖÖÖ,ïÈfÎO9gÇïu2W#ó6l9qnÌ9ölýT$+YÉ*)²ÖÕÕ8qbên|ðÁúõëgCô¾Vçëäädx¹G@¬­­ííí½s÷0È±wîËéêÕ«1?Ù±ÈJVIù%ëÚµk¯>'u¾víÚÆKKK»ººî+kkò=ÔÌ#655%W4<<:ÄÄÜñÅ_~ùåÌÕÈ~ÄÀ>~ÖÔÔ$yi²¬¬Û¶mé¥q^:­­­NT¬©G|åWÂÅ;wJqàÀì/,g_"Æas¹YØßß¸q`` ß1¬d%«¤%k©Gáiò²jjá¿&ÃÁÉÉÉyÉypÄ,ÆåÇD@¼;¯Âøä¨Å«1ÛÇÆÆÚÚÚÆÇÇïÜýÏò+bÕ +YÉ*éAÉjbb¢´´t¬Éá#â¬y½ypÄÄÔ¾¾¾,ÿÿk1k³f<b@ÞÞÞ:Å2>²?YÉ*)¿d]¿~ê¿×<þ|SSS2L1ßÁc±ûÊÚÝÝ]\¼ypÄ;wâ=ztQd¬FæÏ=ÛÜÜOì©eb7ñÂú¿v'+Y%­hY[ZZÖ®]#¹Ð(Û¶mK^V1ßæÍëêê¥Ô%Ì6LÿÖMæÁïÜýß²cÎ7îûyã9ÊLg±ººzÚ÷Ê+jòªiØ"+YÉ*éA½,²¬ÈJV²UYEV²JZ®²Æü'N,àÏÌ#^¼x1y/sùA¬d%«¤yÈ4&_ú¼3ÏÿÀ|ÚÔÎÎÎùåååÌ#+Y%­PY?¾ÿþX÷Îÿ?`ª®®®Ðyd%«¤*küÜ¼yóàà`oÔd!Uêwîª©´´4,óÈJVI+WÖ`5p]À5ý©º»»SÇBYÉ*i%Êíß¿ÿøñãó³N;`zËìd%+Y%Í[ÖwÎø¿ÎVækkk#öõõÅYÌ#+Y%­hY£'NÌ]ÖÌ#444ÄhuË-3dEV²JZþ²¬d%«$²¬dDV¬È*²¬ÈJV²UYEV²J"«ÈJV²JdYÉJVIdYÉ*¬"+Y%Ud%+Y%¬d%«$²¬dDV¬dDV²¬È*²UYEV²J"«ÈJV²J"«ÈJVIdYÉ*¬"+YÉ*¬d%+Y%Ud%«$²¬d%«DV¬dDV¬È*²UYEV²UYÉúY4::º*-²J"«Èº¨N<ÙÞÞnÌJV¬"knV;;;Óç¼ñÆÿxo---kÖ¬!«$²¬÷¯¶¶¶¹¹¹°°°©©ipp0æ;vl×½íØ±¬È*²Î¯ÆÆF¯K"«È³È*¬"ëb_JÆ¬ÍÍÍdDVuQõ÷÷×××nÞ¼9p%«$²¬<²J"«ÈJV²J"+YÉJVIdYÉ*¬"+YÉ*¬d%+Y%Ud%«$²¬dDV¬dDV¬È*²UYEV²UYÉJV²J"«ÈJVIdYÉJV¬"+YÉ*¬"+Y%Ud%«$²¬d%«$²¬dDV¬È*²¬ÈJV²UYEV²J"«ÈJVIdYÉJVIdYÉ*¬"+Y%Ud%+Y%¬d%«$²¬dDV¬dÈJV²¬È*²UYEV²J"«ÈJV²J"«ÈJVIdYÉ*¬"+YÉ*¬d%+Y%Ud%«$²¬dDV¬dDV¬È*²UYEV²UYÉJV²J"«ÈJVIdYÉJVId%+YÉ*¬"+Y%Ud%«$²¬d%«$²¬dDV¬È*²¬ÈJV²UYµ´díîîÎrÇ"«$²¬óhrrrÃ©;Ö§~záÞÎ;WTTDVIdYçÔ/¾øòË/§îXííí«2Z½z5Y%Ud½W¯^mjjJÝ±þð?ÝÛ/~ñcVIdYçTkkë¹sçþ|õÞgDV5×zodDV5gÄ³J"«ÈJV²J"«òRÖ,UYEV²UYÉJV²J"«ÈJVIdYÉJVId%+YÉ*¬"+Y%Ud%«$²¬d%«$²¬dDV¬Èªå.kYYÙ¦Moß¾MV²J"«+kúWYYùøãMMM¬ÈJÖ466600ÐØØXRR®lMMMGGYÉ*¬d]x.µkWº¯q¬dDV²Î£7ovww744§º~ýúªªªÈ¬ÈJÖE½ÏzåÊÉÉÉ8kjj*¸%+Y%¬ó¨¼¼|ûöí>LVIdUîßg%+Y%U9õàÁ1rM¦KJJxâ	²UYÉºÀZZZânQPPþ¶k[[YÉ*¬d]Hq·INÇÉIV²J"+YRrää¹sçâäâ¿lCVIdÕõ­·ÞZÑ±cÇÈJVId%ë;útuuuÑÝªªªººº|¬ÈJÖ<¬È*²¬ÈJÖ644TUUUPPþ>kêK8d%«$²u~UVV¦ä[7dDV².ô7ïÖ××çÕ`²J"«r kyyù½[UYµ²d=sæLÜ-öíÛwëÖ-²UYW§åLdDV².°ò	&²J"+Yó4²J"«ÈJV²J"+YÑÅëêêJJJ·WÈJVId%ëëîîöÁ¥hmm%+Y%¬)9Ó¥KÒeõÿ³UYÉºÐß¼[2²NMMùÖY%U¿]ËÊÊ£& &d%«$²u!uttd)âäÉd%«$²uõöö®[·®¨¨¨°°°ªªêôéÓ>LVId%kFVIdYÉJVId%ëâ>ìüdDVåFÖÔQøÓeuD~²J"+YsÐíÛ·î¹¸tvv¬ÈJÖ)Â÷YÉ*¬dÍ«===Þg%«$²*Ç`Z·nYÉ*¬d]Ô'R«d%«$²5#«$²¬d%«$²5§ï³æðdDV­¸÷Yg5iñ «$²jeÉúòË/ÇÝâ^¿qãÆO>'ßï½¹üîÅ~øáÐ·¡¡¡§§¬È*²Þ)**v·ÅÅÅsùÝ59ZSoooyy9Y%Udý¬Ï<óÌÍ7ÇÆÆ80wYSuuu566ÆÄë¯¿þßïíë_ÿzj±¬lüZKKËó9íO>ñl%UKUÖ#Gd¾ÏzôèÑ9þúäädiiiüJGGG|ûí·¼·oë[kÖ¬YÆ²®[÷åªª¿Ùû/-¹ú;3¶Ú³DV-áoÝ<y²ºº:F1~­ªªúàæ	ÝÝÝ³jxÙ¿·_=üÝ'sx±½dÈª¥-kNíSÄd%«DVu®ÕÖÖ^¼x1&úúúÉJV¬"ë_¾<SWWWRR¢©©i¿800ÐÐÐ£Õ-[¶¬YEÖ?¿E:íK1ÑÚÚ«5#+Y%²jeÉZYYwK.¥ËãW²UYÉº ß¼[2²NMMùÏÉ*¬ZøíZVVw¾¾¾Ôdb¶¯Ð¬ÈJÖûÔÑÑy¤'O¬ÈJÖÖÛÛ»nÝº¢¢¢ÂÂÂªªªÓ§OçpÍÈJV¬Zq²>ÐÈJV¬ZY²VWW×ÖÖ^¾|¬dDVå@Öä>7f%«$²*7²?~<îûöí"+Y%UwÿÖiù>+Y%¬58³Ùþã²UYÉê³ÁdDV¬Èª¥%knßL%+Y%²¬ÿ&ëU¬dÈ*²¬ÈJV²UYEV²U"«¬Yr¤²J"+YçWAÖ)¬ÈJÖ<¬dÈ*²¬ÈJV²UYEV²U"«ÈJV²J"+YÉJVIdYÉ*¬"+YÉ*Ud%+Y%Ud%«$²¬d%«DV¬dDV²¬È*²¬YEV²U"«ÈJV²J"«ÈJVIdYÉJV¬"+YÉ*¬"+Y%Ud%+Y%²¬d%«$²¬dDV¬È*²¬YEV²UYEV²J"«ÈJV²JdYÉJVId%+YÉ*¬"+YÉ*Ud%+Y%²¬d%«$²¬dDV¬dÈ*²¬ÈªÏJÖ¾¾¾6666¬YEÖEU[[ÛÛÛuuu11<<üûÙÏ~VTTDV²JdYçWqqqüloo_ÑêÕ«ÉºldýäOVåºG=;çOq÷ËùMüüóÏUd_ýýýöì7n|zo¿úÕ¯Y¬Õ_ýr·7öÞ?ÿó·xW²æöA·÷_ZÈ*²Î¯±±±¶¶¶ññqï³¬d%«ÈºØFFFÚÛÛGGG6¬d%+YEÖÅvöìÙæææ°3Ë2d%+YÉJVu®UWW§H¬d%+YÉ*²>ðÈJV²¬"+YÉJV²¬d%+YÉ*²Ud%+YÉJV²¬d%+YÉJV²¬d%«ÈJV¬dYÉ*²¬d%+YEV²¬"+YEV²Ud%«ÈJV²¬dYÉJV²¬d%+YÉJV¬"+YÉ*²Ud%+YÉJV²¬d%+YEV²¬d%«ÈJV¬d%+YÉ*²¬d%+YÉ*²¬"+YEV²¬d%«ÈJV²¬d%+YÉJV²¬dYÉJV¬"+YÉJV²Ud%+YÉ*²Ud%+YEV²¬d%+YÉJV¬d%+YÉJV²¬dYÉ*²¬"+YEV²¬d%+YÉJV²Ud%«ÈJV²¬dYÉJV²¬"+YÉJV²¬"+YÉ*²Ud%+YÉJV²¬d%+YÉJV²¬d%«ÈJV¬dYÉ*²¬d%+YEV²¬"+YEV²Ud%«ÈJV²¬dYÉJV²¬d%+YÉJV¬"+YÉ*²Ud%+YÉJV²¬d%+YEV²¬d%«ÈJV¬d%+YÉ*²¬d%+YÉ*²¬"+YEV²¬d%«ÈJV²¬d%+Y<ÕÕÕd%+YÉJV5õôôÔ××g¿W¬d%+YEÖ¹¶ûöË/§ß«^õÕ¯ßÛÖ­[ÉúÊú|NÛ»wï+ÊgYcïåv[ZZb«s~ú)YÜüãçsÝbn²õÚ½êÝwßÝsoO<ñÄ5kÈúÊ7P<µåêßÛöÊ¾ôÅ|5%s»ÉEEÿÿC/0V/þd]pßüæ7ªªþ&n²õÁÊêÕàü5«×ñ¿÷?TVç²Æse×°òË½ó¿öåð«¿úe².¦¸ÃäöA·È[¬d%+YÉJV²Ud%+YÉJV²¬d%+YÉJV5÷¬d%+YEV²¬d%+YÉJV²¬d%«ÈJV²¬dYÉJV²¬d%+YÉJV²¬"+YÉJV²Ud%+YÉJV²¬d%+YÉJV²¬d%+YÉJV¬d%+YÉ*²¬d%+YÉJV²¬d%+YEV²¬d%«ÈJV²¬dYÉJV²¬dYÉJV²¬"+YÉJV²Ud%+YÉJV²Ud%+YÉJV²¬d%+YÉJV¬d%+YÉJV²¬d%+YÉ*²¬d%+YEV²¬d%«ÈJV²¬d%«ÈJV²¬dYÉJV²¬"+YÉJV²¬d%+YÉJV²Ud%+YÉJV²¬d%+YÉJV²¬d%+YÉJV¬d%+YÉ*²¬d%+YEV²¬d%+YEV²¬d%«ÈJV²¬dYÉJV²¬d%+YÉJV²¬"+YÉJV²Ud%+YÉJV²¬d%+YÉJV²¬d%+YÉJV¬d%+YÉ*²¬d%+YÉJV²¬d%+YEV²¬d%«ÈJV²¬d%+YÉJV²¬dYÉJV²¬"+YÉJV²Ud%+YÉJV²Ud%+YÉJV²¬d%+YÉJV¬d%+YÉJV²¬d%+YÉª¥"ëÀÀ@UUUaaaCCÃ3gÈJV²¬"ë¢jkkçwbâèÑ£;wîßüæ7Ý[WW×5k~»Ð~ò¬_ÿÿóÍÕ¿ÍÿðwÿqÃ¿Ïá~å+å»Û¿Ãí­þmîWï»ÿó¢59¼ÀØ­­åp¿ýío?ú¿Ïá~éK_ü×çþ)Xùxhü6_Ëù.n¸Qr¸qÉín·YÉËÊËË§¦¦bbbb¢ºº:&ÚÛÛWeÚ¢¿ðïrW²Jy~«W¯.Ê]¹]½dsi9ÜÞØùÇEõb?ÏåÁø t¹½KîsË«[ä_ü"È³â8múüãÿÍèÖ­[[ü­½nË/|áï¾ûî2ØzèØ±cË`Cªªª9²6¤¦¦æßûÞ2Ø¯íkßùÎw<ïkyÊ§¦ã·Ïv¬d%«´äe­¨¨¸s÷Õà&+YÉJV²¬j×®]ÇøÙÖÖFV²¬dYUOOOeeeAAA<õôõõ¬d%+YEÖåYÉJV²JdÍeßÿþ÷ýë_/yõÕW?ùäe°!¯¿þúÁüà?øå/¹6äG?úÑÏþóe°!o¿ýöGäIOd$¬$uEyàâ¹Êø3¯¯¯oÃ±±Âég¦¾*Ï·(smèM¾éßÒ^B·Èõë×ã Í¶ÿÊM3mCÍEd]2e¸8sNV[[ÛÛÛuuuég<y²½½=û6æOk»ÔoXÃX½%wôôôÔ××§ÿqe%óù¦ÉÜeó`YL.Îç§gÎÎÎìÛ?e®í¾i·lÙrßmÌÃÙ¾ûåËÓAÊ²ù|ÓdnÈ²y°¬K¦ÌgÎÉçúûû÷ìÙ3m8ÛÜÜkÞÔÔ488ç[¹¶Kú¦imm[ä¾Û·RÌÿfFYúEd]2e¸8¯e½±±±¶¶¶ñññÏill[ZÛ¥ÓÄPiãÆsÙÆ¼Ýt²¬dþß4².§Èïe¸8¯eýiº½½tt4Ë2ÉSÃRÙ¢iOdKî¦9tèÐk¯½6mÌÛI)ËJæÿM3MÖå÷`YóºÌçÕ¡gëìÙ³ÍÍÍ×¯_Ï<«¶¶vhh(y6eò|2×véÞ46m:þü¾EÒAÊ²ùÓ¤oÈ²y°¬Kaï>ö2W2­êêêÌ/«$ýýýõõõ7oç<ß¢Ìµ]º7Mz¿L»-¡[$¤Wr©Ü4é²l,"«$Id$Id$¬$U$U$²JDVIDVIÈ*IY%I"«$I"«$Id$¬R®:wîÜÆ·nÝº¨;qÚöÙ<·¹Ú/½ôRMMMå¡C²¯Þ×ùæÍ±XYYY>ìg¬Z)õ÷÷/ÀªªªÔÿ¹k×®e#ëöFNvE°ºêÞyæÅËÚÕÕíØ±¬Yï#¶ä·nÜ¸ÓKZÖE®LNvÅöíÛcÈúôé>þ|¬GÅöïßOV¬úsï¼óNvïÞ=99ÿÁ444ÖÕÕ:uj¶gÛôÉt`ã~øá/¦æ§Ê¯+ûonÞ¼9õêèµk×ùW¯^13YÎ¸Úï¿ÿ~mmm,«+|ßÍâÍ@úbÛuß+qWd¿Þï½ÒÒÒM6Ív»wwwÇbñ»s5>~üxEEÅÚµkß|óÍÔb?þxëZ,îTw;vìDÖU<¦?e÷»ßMæøáÓÍÏ;7GWÒZîkäl×ý·ôs7nÜÌkL?£	*é¥íbç%kæÈ.ë¯4óWî%%¯ÐfWç|1rmÍ§íùÌù	¥Qü'/]º4ãbhYWP<òH2Æº|ùrLÔÔÔ$ócä'zê©ÅÆÏÞ²eË]yúé§§¦¦bÓ1Î2$ïu¥WPPgÍ8¦Lmâòc:péÞ¹sç/6'`Ú%,àJï»ÓöíÛì/-³±LIII²æ.kò¾lüLýÑ[ëCçH-öì³ÏÆtüé6x Id]AÅât1?y?/~fyC.óÉw||ü¾,ìºÒÛ½wÏé6m¡öÍ7SgÅ8ìÀMMM³­@ré4÷½Ø¹Ëzß=0íp¥÷ÝiÙøc¸eWg®óèèhL¤®ñã?éØÛ³-÷14¬+NÖd´±í³fãÊ6B---Éfùo¾ùf<xð`2q­N/TË~±³y³°=0m±ù^éwÚc=C¤äNgo²Æ^&ë'búÉ'L_,yÃ>|M_1Id]%¯>úÒ¥K1±víÚd~òziú©o$,øááñÓO?=/Wnß¾Ï¥Êr]sù é7ÒHÈ¹zõê3gf[·ä½Øîîî¹_læ#ÛÙ÷F+vÉÛiÛ¶mK½Tüøñ|)×±ÕûöíéäãQÉ+Ão¼ñFúbñML§/&¬+¥i`J»1y©0½ÞÞÞä¬Í7g+nÆçå²²²Ù¾åº² üM*õô½~ýúÔÌø9666í¢N<9í·oßýbÓ[ØÈ²7ær¥Ó.ya;­¯¯oÚo½ÿþûó5½äÛ;1Nÿ3%s±ÎÎN4¬+«cÇUTT$ßº¹uëV:ºÉWGêëëÓw/_1_AAAwR0ß×äkqi3²1ÛueAbtt´­­-ùvM¸822ÌïïïO¾òè£Íö=ËX¸®Ø¦Ç¸3õélÞÂö@½1+ÍÜØiwîÕ!V;ßJ±:wYãXóòòò÷Þ/üùºç¤ë&ö­oÝHd4Ë³C@HdDV¬ò´¢»ÙY%I"«$Id$Id$¬$U$U$²JDVIÈ*IÈ*IY%I"«$I"«$I­þfá9Ð,¿"~IEND®B`


 count of sales units for 1.5 Hp/c


9Oø9]A¸&wn­Ï¸	Y_®IÍ4¯cLpÕpÆ¯©+!TcÎ¨F®1+ªÆÂ¸B¨¾/~Ùa¢ÆÒHr5Òó wÞÍÌûÎ~3ï¼zì>°p³@Y@Y@YeeePVPVPV@Y@Ya©?sÈ=¦óLÑÓÓ³uëV«uaÊ:Ë³Ê+½¬ó¼(@YAYg³Þ¹sgïÞ½kÖ¬I¥Rååå/½ôÒØØXú<iÉ¯_¿¾k×®Z[[oÜ¸yçÎ«««+..Þ¶mÛÐÐPö5ÆJKK¯½öZeeeüJæ;;;âÔÍ7_¼x±¾¾>.¼¹¹9N@YaÉuçÎS"úâ/N[Ö[·nEz3gVTT¤#×ÛÛyRMMMö5ÅaKKKÕ)W±eËÌ3gÚ´iSÔ74ÊíeGZÖd5©Ýàà`L=6¦W¯^=m¤_xá8ºcÇ;ÄDÉ©ÑË8ÙÑÑÑt°3/êàÁÉH·ªª*æÄ5ÆôÍ7c:ÚyæûöMLLtuu%Gc`GcÔyN@YaÉuÃ1½~ýúhäéÓ§ïÞ½;Óð7F¨q4F®ÉÑq-++K®Y³&g:åÓ¿²Fnmæ£ÐGoß¾Ófþ²Â¡¬¹çgN$qMD Ï93íE¥R©)GcæL×ÑÅô©1òÞòL.ç>(+,­²&._¾|èÐ¡äÝªªªiÏpÊ5fÎ~Ìyuuu1çå_îììQVPVX&eM>gM>ïñkLg4ö@mooO>gy Éð¾û3·¶¶ÆÑÝ»wß½÷öíÛÛ·oÏ]Öd|õêÕÈ¾÷ÞÊÊË¤¬ÑÂ)oÉîÙ³'9)ÙÈ(´µµÅÑ7n$ÓÌm?ûì³ô¨7ó¤¦¦¦ÜeM§7QVVÉÆÊÊ8p ²²2ÉÛ·¿ýí÷î%'>:âËô7PûûûwíÚUü@R§ltæÌõë×ÇH7NJÞà²¹oæcgËZÇF¶§|áGYAYaEKÞ:îêêéþðq´®®Îeò|Î:Å#G¬PV cccû÷ï_»vmò^qL¼úê«V(+(+ ¬ ¬3úýï`!eý#»ûvQQQÀ²àÃÊº$ÊúÅ/~ñwË²*«²(+Ê ¬(«²Ê²*+²¢¬Ê²z6Ê²*+²*«²(+Ê ¬(«²Ê²*+²¢¬Ê²*+ ¬(«²(«²*+²¢¬Ê²þK&«««ÓG«ªªR©TýùóçPVuºººêêê2U­­­ÇwÞyg÷îÝ1ñßü¦ÿó>ýôÓââbÏF@YQÖ©¶oß>00ù¨*//ñññd,ÛÞÞþXÇÜ³PVuëÎxT¥R©)ÓÆ¬²¢¬ùµ¨¨(=ùô9+ ¬(ë¼ÊZQQ1>>¼ÓÊ¬Ø²nÜ¸1Ùî$qòäÉ³t¶*½páB]]]rRWWWòb9LRÖ?rYÛÚÚ;qØÚÚª¬À-kÌNLLÄtÖÖÖ.öèv6[fn388qã¿çWÖ¥RÖ¸_+++ãø+©»»[Y7ÞxãÌ31û÷ïOÎ9<<¼eË,FwoÜ¸,cNEEEmß=räÈºuëbæ'|²[¦­_¿~hh(&â0¦ÓóoÞ¼7OVïÛSÀ,kTjïÞ½11ØHÎ¹k×®ÎÎÎ¸xñbÒ°hÆtL~½´´4ùÝ7ß|3¢YÍÜ2tÊ¶Ó^uî­J3ß(.))3ÇaOOOz~KKKæQeUV%TÖ8Ü¹sg644¤ç§»ÔîÎ;1BmkkK¿ÉXsJ)p«Ò1_»v-Iì¦M1äMO£¬K±¬o½õÖ©S§öìÙYÖ÷îe3bÖÑÑqáÂ[·n¥Ë:m)ç:fÍ±Ué´ÃÙ×^-n°¦*+ÀÒ-ëÐÐP$-ù´5óÌ3Ï>|8&¢¦O?ýtÒÚë×¯íÝ»÷¡e]¨­J#ç1qõêÕôFËMMM/_ÖTeXºe[·nM¶N·65mhh9|ðÁêÕ«KKKß÷Ý-ë´[&gGP©b:ýqú]h`ieUVeEYeUV`%µ¿¿ëÖ­ÅÅÅ%%%ÍÍÍÉW2:ífÀsÚ_D~/Ëûióã¾¾¾äÓÙô>»»»7lØsz§£¬Ê°e­­­=uêÔäüñºuëfèC·¬F)®ÙûDìèèhooöÌÔÓ§OÇÄ¥KÊËËc¢¦¦&¦ï?Ø1r,ò´sUY ¬¥¥¥émnÓ[Þ¦G·nÝÚ´iÓ5kÎ;÷Ð²&qM¾u½sÄÆÆÆänÞ¼YYY9×³÷YMòCÜìd÷"ÿ£¬ÊgY#NÛ¶mã71,[ZZ9MYÓG³wøæo=zôþ½R<x0÷Ë³Ù¿D:#ÞÑìþþþ)ç?âüÉ.Ózzz]9æ£¬ÊYC£©o¿ývOÿ9>sú£ª95çCCCÉ.	#´É§¹ùTdºÛ=0MDÝ3wê4::ÚÚÚ:66c²*+À|Ë6>>ã¼)eMv'ÍéÝàì#&MíîîÎñÿ_ç4fòöLÞ'b¸½½xx83ÉSæ(«²Ì·¬ëÖ­Kv±._¾ÜØØxÿÁ.ò.ÆxîÐ¡C7oÞ³=´¬1@,))IÞÎÞ9âýq3ÏçªNy78ù7ÙÜÜy¶8)Ù'b<9éÂ1/ìéódÏQVeX²ö÷÷ïØ±£´´4ÆvQhÌÜ¶m[2ñÜæÍkkk#TéKiçûßºÉÞ9b9wîÜyèt6eíéé©««+[ü×ôzëëëã¤%'CÒêêê)£áì9Êª¬ün0Êª¬Êª¬Ê ¬(+Àr-kÌ?uêT|fï0(«²+±¬Æô:g_Öì½fïSeUV`%õØ±cÈocÝÄ½N»OAUYRÖ8Ü¼ys²À¾Qã«)i(«²+«¬ÕkcÖi÷8e(«²+®¬áÀÇÓ5ÇÞÓûDYX¡eqçf?fÍÞ;`ö>QVeVnYÃ©S§f_Öì½fïSeUV`ÅeUVeEYeUV@YQVePVe]¦eýå/ùOÄgæ%PVe]þeåWâqüµMõúW	÷(«²®²îû;®ýßõ§ú+ÿZYeUVeUV@YQVeeUVeUVeUV@YQVeeUV@YQVeUV@YQVeeUV@YQVePVeUVeeUV@YQVeeUVeeUV@YQVeeUVeUYUYeEYPVUYUYUYeEYPVUYeEYUYeEYPVUYeEY@YUYPVUYeEYPVUYPVUYeEYPVUYPVe].PWWJ¥êëë»ººPVu^ÊËËc"«««PVu^Ö¯_?44qÓ1ñ½ïïéÏÛ¸qcjPVõázKJJâq===1çÇ?þñÿú¼ûö­ZµJYeEY.Æ£×®]K»iÓ&ïÊ²ÎK*vZYeEYóãÔ¾¾¾¸zõj_PVu^®_¿l 1­¬²¢¬NYeEY@YUYPVUYeEYPVUYPVUYeEYPVUYPVeUVeeUV@YQVePVeUVeeUV@YQVeeUVeeUV@YQVeeUVeUVeUV@YQVeeUV@YQVeUV@YQVeeUV@YQVeUV@YUYPVUYeEY@YUYPVUYeEYPVUYPVuQR©ÇÿìÏJzªjñ~WýëM^@YQÖQÖ/¯:þ£ýçÿéÈâýyýùÿÖ«(+Êº"ÊúÄ%¿E½(wSÓ×¼*²¢¬Êª¬²*«²*+ ¬(«²Ê²*«²²¢¬Êª¬²¢¬Ê(+Êª¬ÊÊ²*«²Êª¬Êª¬²¢¬Ê(+Êª¬ÊÊ²*«²Ê²*+ ¬(«²*+(+Êª¬Ê(+Êª¬²¢¬Êª¬ ¬(«²*+ ¬Êª¬Ê(+Êª¬²¢¬Êª¬ ¬(«²*+ ¬(«²Ê²*«²²¢¬Êª¬²*«²*+ ¬(«²ÊÊr(kYYYSSSÿ½÷UYee¾e,Ceeå³Ï>ãÆÉÉIeUV@Y5£££½½½«W¯Î¬ìúõëO<©¬Ê(«²æïêÕ«mmm£Êª¬²*ëtvvÖ××d6uÝºuUUU1UVeUYçõ9ëõë×'&&â¤ÉÉÉÈ­²*+ ¬Ê:åååÛ·o·m°²ÊÊÂÎ:çöööâââuëÖ]¸pAYXÖCÅÈ5^½zõsÏ=÷E½öÚkG¬®_¿>ætuuýèóÞzë­U«V)«²²²<ËºcÇxXe~ìÚÚÚß¥Õ××_»v-sÎ·¾õ­ÒÏòÉ'S©²*+(+Ë³¬¹xX%GoÞ¼GcfÞCÒ°^¹rÅ»ÁÊÊÊ+k²®®®äèÅãhÞ_¶±ïøÃèïïollTVeeeÅ5BøX>ø ¿K«¨¨È¿*«²²²·`:öluuuñUUUçÎËû¢öíÛ÷ÑGÅD__ßÓO?­¬ÊÊÊJ,ëiiiÑjccãÀÀ²*+(+ÊºèUYAYYÎe½qãFUUUQQQæç¬é/á(«²Êª¬sSYYÔDÞßºQVeee¥5ijww·wPV ¬åååøa¡¬ÊÊÊr.ëùóçãa±ÿþ»wï*«²ÊÊÂ¼<-PVeÍSÑtlÁ¤¬²*kÁPVeeEYUYeUÖYëëë«­­]½zuòñêLÿ£FYPVe¸ÎÎÎ).ÅDKK²*+ ¬ÊdL×®]Ë,kÞÿUY^Öd´LDY'''ëFYe%ÿûµ¬¬,Ù»aÔd"ó+«²Êª¬spòäÉì=Ettt(«²Êª¬yºtéÒÚµkS©TUUÕÙ³gm¬¬²*kÁPVeeEYUYeUÖ¹llüÊ(+SÖô^ø3ËjüÊ(«².÷î½üòËñ(9ú´²*+ ¬Êº=Eø>«²Êª¬Õ®®.³*+ ¬,ðLk×®UVeUYçµSZdu||PVe-Êª¬ ¬(«²*+ ¬Ê:ÏYuÊª¬ ¬,óÏY§jb1v¡¬ÊÊÊr.ëÑ£Gãañê«¯Ý¹sçùç£'Nðn°²Êª¬ù(..ò°£%%%Êª¬²*kþeé¥FFFFGG<¨¬Ê(+ùß¯o¿ývöç¬ï¼ó²*+ ¬Ê§êêêT*ã×ªªª?þxQo«²*+(+Ë¼¬²*+(+Êª¬Ê(«²ÎZ___mmíêÕ«ýB466*«²Êª¬yêìì²Ç¥hiiQVeUYóQYYk×®e5Æ¯Êª¬²*k^¿ù@2eôÏPVò¿_ËÊÊâaÑÝÝ5¨¨¨PVeUYóqòäÉì=Ettt(«²Êª¬yºtéÒÚµkS©TUUÕÙ³gm¬¬²*kÁPVeee9µººº¦¦f``@YPV ¬É>7fUV@YY²;v,û÷ïTVeûóÿ>ë¾Ïª¬²*kþïgK¥RÊª¬²*«mPVUYeeu±?LUVeeeåõWVYeUVeUYUYeEYPVµ¬9ØS²Êª¬sS=E(+ ¬ÊZ0UYAYQVeUV@YUYPVUYeEYUYAYQVeUV@YQVeeUVeeEYUYeUVeUV@YYeíììÌñPSVeeEYç`bbbÃéÚþðÉÏûì³ÏUYAYQÖÙ:|øðÑ£GÓµöööìÿ¥óøã+«²²¢¬788ØØØÓôCíÊ+§?ïÃ?,..VVeeEY®¥¥åâÅ÷üÿW³*+(+Ê:ïÛñyÊª¬ ¬(ë%ÖUYAYQVeUV@YYeÍAYeUVeUYUYeEYPVUYeUVeeUV@YQVeUVPVUYPVeUVeeUV@YQVeUV@YUYPVUYeEYUYAYQVeUV@YQVeeUVeeEYUYeUVeUV@YQVeeUVeeEYUYeEYPVUYeUVeUYUYeEYÕ«(+Êª¬Ê(«²*«²Ê²*+ ¬(«²*+(+Êª¬Ê(+Êª¬²¢¬Êª¬ ¬(«²*+ ¬Êª¬Ê(+Êª¬²¢¬Êª¬ ¬(«²*+ ¬(«²Ê²*«²²¢¬Êª¬²¢¬Ê(+Êª¬ÊÊ²*«²Êª¬Êª¬²¢¬Ê(+Êª¬ÊÊ²*«²Ê²*+ ¬(«²*+(+Êª¬Ê(«²*«²Ê²*+ ¬(«²*+(«²*«²*+ ¬(«²Ê²*«²²¢¬Êª¬²¢¬Ê(+Êª¬ÊÊ²*«²Êª¬Êª¬²¢¬Ê(+Êª¬ÊÊ²*«²Ê²*+ ¬(«²*+(+Ê:WÝÝÝ6lH¥R½½½Êª¬ ¬(ë¼ÔÔÔt)&N<Y[[«¬ÊÊ².8<vìXûçµµµ­ZµJYÿáûß|ê©¯¼²ø>ýôS¯ ¬|YzzöîÝ?øÁþÓçmÛ¶-J)ëwì'd,Ë¢þÄU¼ÿþû^û@Y)ì²¶¶¶y78wYëÿjÝb/È®¯¬ ¬vYÚÛÛm¬¬ ¬(ë|]¸p¡¹¹9Úã<Êª¬ ¬(ëlUWW?AYe]tÊª¬ ¬(«²*+ ¬Êª¬Êª¬ ¬(«²*+(+Êª¬ÊÊ²*«²Ê²*«²²¢¬Êª¬ ¬(«²*+ ¬Êª¬Êª¬ ¬(«²*+(+Êª¬Ê(«²*«²Ê²*«²²¢¬Êª¬ ¬(«²*+ ¬(«²*+(+Êª¬ÊÊ²*«²Êª¬Êª¬ÊÊ²*«²²¢¬Êª¬ ¬(«²*+ ¬(«²*+(+Êª¬ÊÊ²*«²Êª¬Êª¬ÊÊ²*«²²¢¬Êª¬²*«²*+ ¬(«²*+(+Êª¬ÊÊ²*«²Ê²*ël~þªn]UUeSÓ×õgíÚ§ÇKä³Ï¶ýõ_×-êºú¿©ÿÓ?-Yìä©§¾òþ¢úú×6nüÛE]¿üË'|b¯.eUVe]Yeý¿ørËùwÇ´Qâey5²®E]W¾ó_cu-ö=òw_ý·ñLYìÕUêÈëÏ/ê<÷lóÒ_]Êª¬Êº²ÊZóo¾¯-Ë¦¬1|×ÙE]W'ÿñÀ¿*[³Ø÷H<GAYzªj±&ÿðýo~éKO.ñÕ¥¬Êª¬Êª¬Êª¬Ê²*«²*«²*+Êª¬Êª¬Êª¬(«²*«²*«²*+Êª¬Êª¬Êª¬(«²*«²*«²¢¬Êª¬Êª¬Êª¬Êª¬Êª¬Êª¬Ê²*«²*«²*+Êª¬Êª¬Êª¬(«²*«²*«²*+Êª¬Êª¬Êª¬(«²*«²*«²¢¬Êª¬Êª¬Êª¬Êª¬Êª¬Êª¬Ê²*«²*«²*+Êª¬Êª¬Êª¬Êª¬Êª¬Êª¬Êª¬(«²*«²*«²¢¬Êª¬Êª¬Ê²*«²*«²*«²¢¬Êª¬Êª¬Ê²*«²*«²*+Êª¬Êª¬Êª¬Êª¬Êª¬Êª¬Êª¬(«²*«²*«²¢¬Êª¬Êª¬Ê²*«²*«²*«²¢¬Êª¬Êª¬Ê²*«²*«²*+Êª¬Êª¬Êª¬Êª¬Êª¬Êª¬Êª¬(«²*«²*«²¢¬Êª¬Êª¬Êª¬Êºøz«ªªR©TýùóçUYUYeÖÖÖãÇÇÄ;ï¼³÷îøõ¯ýéç;wnÕªUÿ¯ï|ç;ÏüÇ¿û??þûEý).N½ûö¾E½çþûøråA¾üåò=íßXìkW^6lhøûÿÛ¢®«×ÿ÷sÅ«RÄs$)½ºÊÊJûiòâÿüÏñd«KYuqONNÆÄøøxuuuL´··?%µÅùzüñÇãþd=«XN×2§;1EEEÅKÏ¾ðeóègÊYsbþÉâdà¹®®LO<ñ)ë"gæéßýîwÿ/ËÝ»wå­úó?ÿó7ß|sÜÍßüæ7¿ño,éèè-ùío/Ê?ûÙÏÁ²466¾òÊ+Ë`A¾ûÝï~õ«_UOYc ?åÈ­RVeUVeB-kEEÅøøøýïÇ´²*«²*«²¢¬óÒÖÖvìØ±ÃÖÖVeUVeUVeEYç¥«««²²²¨¨¨ªªª»»[YUYUYQÖeHYUYu!½ÿþû¿øÅ/Áüô§?ýÉO~²äW¿úÕÑ£GÁÜ»wïûßÿþààà2X?ü°««k,ÈÏþó'NxÝCY@Y@YWì=ÏfÆoßNvÓ"ÄÒuwwoØ°!nICCCÜªÂ]¾¾¾7&·$y´@$ÑÙÙÞ	_á.ÈððpæÞÜýNAYLö³ç,)ñÚ]WW¹Ò7x)/]MMÍ¥KbâäÉµµµ» ñâúôéÅ)///Ü	ñçNúÑU¸ÒÑÑÑÞÞûîÊºÔeïÁ8Î²ûöÌ²æ¸Á²t%%%Ë`AÎ;ãï^Ã=z4ýè*Ü¬&î,§§	ÊZ0²÷`=g)ÞeÍqbézzzöîÝ[ÐC½5kÖÄãïÂ]ÁÁÁÆÆÆ(JúÑU¸÷HMMMsssÜX¢þþþeð4AYIöæ>s5Ç^úK7::ÚÚÚ:66VèrÿÁ'É®:tAZZZ.^¼ùè*ô$%o$,eAYFöæ>s5Ç^âK¯zíííÃÃÃ¾ SÆ7º Sþãò¸GÒiË²ì=/Íç(k¼îÂÍÍÍ·oß.ô©©©éëë»ÿ`kçX¢Â]ìGWá.HÜ)7nÜHþz[wÊZ ëâÁËGöæ>suÚ¼ô®ººzÊ©@¤···¾¾>F«[¶lIÆßº Ù®Â]ººº¸S6oÞq]wÊÊ(+(+(+ ¬ ¬ ¬ ¬²²²ÊÊÊ³qñâÅM6¥R©­[·Îëû/ÿiîöÌßXÀUÆÆÆÊËË§½=÷îÝ,Ë,gË822g+++óe%===yô ªª*ýâÞÖÖ¶lÊÇÚXÀU111ÑÒÒ2Óéìì²;w.Î¶k×.ÏPVuØßºsçNLtYçycjUr¥±±1G2;óÛÛÛóXY.×Ûo¿g;pàç(ëòtüøñïÙ³'F3éùüq*ª­­=sæÌL¯Gé444Ä/nÜ¸±¯¯/=?Ç«ù´×û·úûû7oÞ~wôÖ­[ÉüÁÁÁ83£ÃioöGTSSç7ø¡£³YgË^®^é´«"÷tâÄ5kÖ455MQ±ø3ÝÏ?ÿ|Ì¯®®Deeå»ï¾_YéètEEEiiéï½>Û³Ï>'ÅíÏýe¥ Å«[æKöë¯¿ÌÿäO¦¼_¼xq]Éiyh#gº®Ü¿QÉ<uÓ¦MÉü¸ÆÌùQìÛýg:À3]ìÊ½rõ¡Wý+½Ùï¸Æwwwç_FÚ§ÇL+aZ3!Òø³#^»v-Ç#BõôÓO'c¬X¿~æËë/¼c8é-[¶Ì²+/¾øâääd¢b:"98s½®LEEEqÒ´cÊÄÍ7gºqù1qé®®®Þ½÷,/vAÖÀKÈãJºÒöïß¬Y^`ÚÎ;cXÃgþqGY_zé¥ÃôåÄÅÆòÆP;&r<AY)Tñ7íËkô æ'çÅaLÌ²+cccmI~×iÏ=qR¼F755Å@gdd$ÒåË<þ41û¢kÌ´fÍ^ììËúÐ50åò¸Ò®´Ü¾?»ÏD#~qxÌò×³qxx8¦Ò·ðÊ+1÷NîG (+]ÖdôGífÆ¢5Æj¡;v$Í677'óßï½8zèÐ¡d4íH:ST-÷ÅÎÔüÖÀ³ÍõJó^i³)ë3Ï<g$uyÉy5î)e=uêTL?ÿüó¹ ¬ªä½¸³gÏ^»v-&JKKùÉû¥o6¦¿%¼~òÉ'ñjøâ/Î©+÷îÝKçÒrl"qçÎÌqUÁÁÁóçÏÏtÛÏb;;;g±Ùä·r¯W:åç¹ÒrmûöíéwqãÞ¹sgÞeáx¬¥ýû÷Çt²9UòÎpzÃ¨ ¬ª)Û$¯§!ù0Ó¥K6oÞû£µi_gËÊÊfú.fëÊä9-½ìºuëÒ3W¯^£££S.ª££cÊ5FNr_l¦üÖ@µ1+rÉù­´>3e4õêÕ¼Ë)òyÿÁç¸ÖÌôe¥ðÁÉwîÞ½Ýä«#uuuÃ»óÅnmmmúeñ¡]9~üxeee´ÙéºrDbxx¸µµ5ùvMtqhh(ßÓÓ|åg¹qãFúS.*nO,EbÜþhs¦ÍßÈ±6fs¥Ù«"6²Ó§O×ÔÔÄnØ°!þ³òf/oüKZ^^~âÄd~òçNæ#m¦G (+Àý¹PV@YAYG®øëPVPVPVPV@Y@Y@YeeeeÂÿÙóÿ7XÓIEND®B`


 count of sales units for 2.25 HP/c


îÜ¹.--é^z)®îØ±ãÞ£b"®ÆÌäÖð2®Æ@vtttdd$væª<899t«ªªbNÜcLß¼y3¦ÃÎÌ÷íÛ711ÑÓÓuQoæÈ*¬1½víÚ0²³³óþýû3cWcäãêªU««+W®«wîÜÉ¼uÊ=¦¿6i||<dnmæÂ!tæÕ»wïÆtÀìÝ_¬Ò_AÖÜó3§È³gÏN»ªT*5eÍq5fÎt¿Ù÷.¦oÁè×g:t9÷UIdKÖ¤+W®:t(yE·ªªjÚÅ§YcæÜÇ¬÷XWWs^õÕ®®®²JdY÷Y÷;cüÓÅÅÅ7=*®¶··'ï³<*axß¾ÉÂ­­­qu÷îÝ÷ïß¿÷î¶mÛrË¯]»Ùwß¬Y¥§DÖ°pÊK²öìInJ2ÚÚÚâê7iæ±ÁþyzÔyÓÆsË¦7iÕªUqiLV¬Ò5Æ£¨¬¬LxûÁ~ðàÁä¦ÎÎÎÀ5éO îÚµ«øQ1HrDÒÙ³g×®]#Ý¸)ywÊá¾Ç¸6õÖq§ÁöüU"«´¬K^:îééé'NÄÕºº:[F"«¤|JÞgÒ#Gl¬òilllÿþý«W¯N^+×^ÍfÈ*IY%IY%I"ëñÅô!²þ»÷nQQQ$=ùÄ0YBÖçîÏôTDV²UÈ*²JYEV²J"«ÈJVI"«È*IdYýoDV¬DV²UÈ*²JYEV²J"«ÈJVI"«È*IdYÉ*¬"+Y%¬d%«$Ud$²¬ÿÊduuuúêåË«ªªR©TýÈ*¬"ë<êéé©««ËÜ«Z[[O:Çß½wL|öÙg÷»ßý®¸¸ØÿFIdY§¶mÛ¶¡¡¡Ì½ª¼¼|rr2&ÆÇÇ±lû3Y=ûì³þ7J"«È:ÃgìU©TjÊ´1«$²¬ùËZTT>½Ï*¬"ëd­¨¨O^i²JZ¶²®[·.9î$©££#æ<ÑgãîîîºººäÒÌbÀ³k×®iAUÊÚÖÖvòäÉËÖÖV²JZ¶²Æü tbb"¦ã²¶¶öInËËËoÝºq9Ìx$½½½1100°oß¾iAUÊ?ªÊÊÊ¢¢¢ªªªä§HVIËVÖ7ß|óìÙ³1û÷ïO¼sçÎæÍcdÚÝ¸qãá£Ï+Æ2æTTTÄÐ6ùÚ#G¬Y³&f~ôÑGs|6^»víððpLÄeLgÞyLYYÙ´ ªdod´Ld½yóæÞ½c:.c°,¹k×®®®®¸xñb1vé¸~ýzÂ^,ùÖ[oMNN«(&7eyS]RR3ã²¯¯/ó¦þþþxÿý÷3WHV²JÒR5.wîÜÀS\v1AîÞ½1BmkkK¿<É'ç_b×¯_yÓÕ«WkkkCÜ£GÆ%YÉ*IKUÖ·ß~ûôéÓöìÉõÁKgÎéîî¾ûvZÖðË1fÍþÜcv1,¿ÉJVIZª²WTT$ï¶&s¶oß~øðáM7lØXàíÝ»wVYsB'/ù^»vmÊqÈ5551qpYßxã²Uª¬Ñ-[#ÓÖ¨¡iCCÃÐÐPÌyï½÷JKKËÊÊÞyçÈ<¨É±Q1ùå1 ^½zuÜià<²U¬"+Y%¬"«$Ud%«¤å ëààà-[KJJÏÃ´´´äxG6~úÃ9s?_D~OËsüKÛýýýÉ[¹é(f7±···±±1ihhU¬´8²ÖÖÖ>zòQ~øá5kfBtÖc Õàù	á:¿´.@íììK.?î¼555qëÃG§J@V²JÒâÈZVV>@7)¾ûöúõëW®þüùYeMpM>¢rÄ2&wtóæÍÊÊÊô)&æØÒvvñ°Ó_d71³Ì³R¬´ YclWQQ±uëÖ7ß|3ÃebÙÒÒräÈ8|øð_Í>9â[o½uôèÑÎJqðàÁÜ/,g_"û¾rqbbb"~!åSÏtÞÄ¨¯¯/9¹#YÉ*I k#¿0õØ±c1<M^_M/ã×dPÍKÖì#'ç2hwsóQdi;tOþTèLçMmmm#+Y%iÑdM7>>ã¼)²&glæõjpöÉSsüý×yYçò¶³³çMìÛÛÛïÜ¹ãØ`²JÒ¢ÉºfÍäKÑ+Wá`âbç:tóæÍXlVYcÃÁäEàì#>|t§Xøøñãù+2·¿´]SS¼ö777?î¼ÝÝÝqS<ÕûÔY%i1eÜ±cGYYYíB@4fnÝº5y5Æs6mª­­¨ÒkéäûºÉ>9b422sîÝ»7ëñÆsuÚ¿´,Ö××ÇãQr2$Í>obuuuîñ1YÉ*I5Xd%«$¬d$²¬ô´ÊóO>ÇÙgÌ>§ ÈJVIËQÖ 1ýÎ¹ËvÀìs¬d´e=yòäò;X7iÊÙÓçYÉ*i9Ê6m|8ó'jr|4%óìSÎ)(²UÒ25X³NvÀô9EV²JZ¦²F8yòä¼Æ¬9Î|¬¬1îlllû5ûìÙçYÉ*iùÊ>zî²f0û"+Y%-;YEV²JYEVI"«ÈJVIdYÉ*Id%+Yÿ]¹rå×Ýçî©P"«ÈºdúÎw¾ÿÍ¾µ¾¾0ÿÅcûøã=JdYLßýî?yãÅy¯0ÿUãkdÈ*²¬YEV²U"«ÈJV²J"«ÈJV²JdYÉJV¬"+YÉ*¬d%+YÉ*Ud%+Y%²¬d%«DV¬dDV¬dÈ*²¬YEV²UYÉJV²U"«ÈJV²JdYÉJVId%+YÉJV¬"+YÉ*Ud%+Y%²¬d%«$²¬d%«DV¬dÈ*²¬ÈJV²¬YEV²U"«ÈJV²JdYÉJVIdYÉJV¬"+YÉ*Ud%+Y%¬d%+Y%²¬d%«DV¬dDV²¬dÈª¥&kwww]]]*ª¯¯ïéé!+Y%²¬ª¼¼üÖ­[1ÕÕÕd%«DVuA­]»vxx8&â2¦câÇ?þñÇ[·n]jÉJV¬"ëì]¾|¹¤¤$ö³¸ìëë9¿úÕ¯þçãíÛ·oÅd%«DVuöb<:00»~ýz¯U"«Èº R©Ô´Ód%«DV5bÚßß×®]ñ+YÉ*Ud]P×¯_OPË&+Y%²¬O<²U"«ÈJV²J"+YÉJV²JdYÉJV¬"+YÉ*Ud%+Y%Ud%+Y%²¬d%«DV¬dDV²¬dÈ*²¬YEV²UYÉJV²U"«ÈJV²JdYÉJV¬"+YÉ*¬"+YÉ*Ud%+Y%²¬d%«$²¬d%«DV¬dÈ*²¬YEV²UYEV²U"«ÈJV²JdYÉJVId%+YÉJV¬"+YÉ*Ud%+Y%¬d%+Y%²¬d%«DV¬dÈ*²¬È*²¬YEV²U"«ÈJV²J"+YÉJV²JdYÉJV¬"+YÉ*Ud%+Y%Ud%+Y%²¬d%«DV¬dDV¬dÈ*²¬YEV²UYÉJV²U"«ÈJV²JdYÉJV¬"+YÉ*¬"+YÉ*Ud%+Y%²¬d%«$²¬d%«DV¬dÈ*²¬YÉJV²UYEV²U"«ÈJV²JdYÉJVIdYÉJV¬"+YÉ*Ud%+Y%¬¹[µjÕÆ<x@V²J"«*ë3UVV¾ðÂ7nÜ$+Y%¬ù4::zùòåÒÒÒLe×®]ÛÑÑAV²J"+YóïÚµkmmm¾ÆU²UYÉ:FFFºººêëëKJJ2M]³fMUUUdÉJVId%ëÞg½~ýúÄÄDÜ499Ü¬ÈJÖyT^^¾mÛ6ÇUYµøï³.¼à¹½½½¸¸xÍ5ÝÝÝd%«DV-GY:#×dº´´ôßû^Þ«zýõ×9299¬®]»6æôôôüÓã½ýöÛ+V¬È'~]ÀýÃ?4U"«µ¬;vìÝ¢¨¨(óm×ÖÖÖüÖV__?009çûßÿ~Ùãõ«_M¥RyïÄ?úÑâ~kaþûÊWÉ*UËZÖ@.váááäêÍ7ãjÌÌm1$-))ëÕ«WÄ«Á!ë¾ÿ±£`éªùæ7È*UËZÖä===ÉÕ/ÆÕ¼?lcß'NÄÄàà`SSYÉ*UËNÖð¬Þï½üÖVQQ9~%+Y%²j9ÁtîÜ¹êêêâGUUU?>ïUíÛ·ï>þþþ6¬Yµe]ÄFFFZZZb´ÚÔÔ444DV²JdYxd%«DV=Í²Þ¸q£ªªª¨¨(óÖôpÈJVId%ëüª¬¬Ì5)ïOÝ¬YµÜeMLíííõj0Y%U kyyù¼[¬Yõ4ËzáÂØ-öïßÿþ²UYµ8¯OÉLdDV²æYÑt9¬ÈJÖ%YÉ*Ud%+Y%¬s®¿¿¿¶¶¶´´4yu¦¿QCV²J"+Yg¯««kÊK1ÑÒÒBV²J"+Yó)9ÓÀÀ@¦¬yÿV²U"«»¬Éh5Y'''ê¬Èªü®«V­JÎnLdþs²UYÉ::::²ÏqæÌ²UYÉg.]Z½zuqqq*ªªª:wîcÉ*¬d]2¬YEV²UYÉ:c¬ÈªÅ5þLY¬ÈJÖEèÁ¯¾újì%d%«$²uJÎáó¬dDV².«===Þg%«$²j`Z½z5YÉ*¬d]ÐLéÕññq²UYÉº4"+Y%²¬d%«$²uï³>ÑSF¬Yõ¿Ï:-¨IOâd%«DV=Í²=z4v×^mllìÞ½/¾øb}ÿý÷½LVId%k>OÙ-âjII	YÉ*¬dÍ_ÖW^yedddttôàÁd%«$²*ÿë±cÇ²ßg=~ü8YÉ*¬dÍ³3gÎTWW§R©¿VUUøáOô±¬YõËú%GV²JdYÉJVId%ëëïï¯­­---MÎÑÔÔDV²J"+Yó¬««kÊb¢¥¥¬dDV²æSeeeì²Æø¬dDV²æõJ&BÖÉÉIù¬Èªü®«V­Ý¢··75¨¨¨ +Y%¬ùÔÑÑ¦3gÎ¬ÈJÖ<»téÒêÕ«S©TUUÕ¹sçLVId%ë¬dÈª§YÖêêê¡¡!²UYµ²&ùÜ¬ÈªÅõäÉ±[ìß¿tttrr¬dDV=çY§äó¬dDV²æÿjpv©T¬dDV²:6¬dÈ*²¬YõÈú¤ßL%+Y%²jùÊú%+KV²JdYÉJVId%+YÉJV¬"+YÉ*UKUÖ9SY%¬ó«(gÎAVId%ë¬dÈ*²¬ÈJV²¬YEV²U"«ÈJV²JdYÉJVIdYÉJV¬"+YÉ*Ud%+Y%¬d%+Y%²êéµ««+Ç®FV²JdYçÑÄÄDccczWûË_þ2ùxþ9YÉ*Udk>zôhzWkooÏþ[:Ï>û,YÉ*Ud½[·n555ÅÀ4½«]½zµóñ~ñ_¬YEÖÙkii¹xñâÃGÿÕû¬dÈ*².øq<YÉ*Ud]4bYÉ*Ud%+Y%U)kÈJV¬"+YÉ*¬d%+YÉ*Ud%+Y%²¬d%«DV¬dDV¬dÈ*²¬YEV²UYÉJV²U"«ÈJV²JdYÉJVId%+YÉJV¬"+YÉ*Ud%+Y%²¬d%«$²¬d%«DV¬dÈ*²¬ÈJV²¬YEV²U"«ÈJV²JdYÉJVIdYÉJV¬"+YÉ*Ud%+Y%¬d%+Y%²¬d%«DV¬dDV²¬dÈ*²¬YEV²U"«ÈJÖe#ë×+Ëóß|y~*ûì³Ï>.ìþð?Ud%k>ÿâ)à+_y.F®ù/Ê~þóÇ·w¼xb!«ÈJÖ|þ=ÿ|É_)dø!ô´Êº«å?ìO)dYÉJV¬"+YÉJV²Ud%+YÉ*²¬d%+YEV²¬d%+YÉJV¬d%«ÈJV²¬dYÉ*²¬"+YEV²¬"«ÈJV²Ud%«ÈJV²¬dYÉJV²¬"+YÉJV¬d%+YÉ*²Ud%+YEV²¬d%+YEV¬d%«ÈJV¬dYÉ*²¬d%+YEV²¬"+YÉJV²Ud%«ÈJV²¬dYÉJV²¬d%+YÉJV¬"+YÉ*²Ud%+YÉ*²¬d%+YEV²¬d%«ÈJV¬d%+YÉ*²¬dYÉJV²¬"+YEV²Ud%«ÈJV²UdYÉJV²¬dUaËÚÛÛÛØØJ¥._¾LV²UdYTMMÍ¥Kb¢£££¶¶¬d%«È*².Z%%%qyòäÉöÇkkk[±bYÉJV²UdG÷îýìgßy¼­[·¦R)²¬d%«È:×FGG[[[ÇÆÆ¼LV²¬"ëBnoo¿sçcÉJVUd]hÝÝÝÍÍÍageÈJV²¬"ë®®~&#²¬"«ÈúÄ#+YÉJV²¬d%+YEV²¬d%«ÈJV¬dYÉ*²¬dYEV²¬"+YEV²Ud%«ÈJV²¬dYÉJV²¬d%+YÉJV¬"+YÉJV²Ud%+YÉ*²¬d%+YEV²¬d%«ÈJV¬d%«È*²¬dYÉ*²¬"+YEV²¬d%«ÈJV²Ud%+YÉJV²¬dYÉJV¬"+YÉJVUd%+YÉ*²Ud%+YEV²¬d%+YEV¬d%«ÈJV²¬dYÉ*²¬d%+YEV²¬"+YÉJV²Ud%«ÈJV²¬dYÉJV²¬"+YÉJV¬"+YÉúevýúõ¿ýÛê¿Uÿ¾ùÍ÷Õ¯>_°ïkÿvÇöoUd%+YÉúÿûøããáú§ýù¯eçÒ¯<W°¯©éÛ¿ýwdYÉJV²>&kõ7¾V°îØ]ÿÖìÃûïÿíï¿óíõdYÉJV²¬d%+YÉJV²¬"+YÉJV²Ud%+YÉJV²¬d%+YÉJV²¬d%+YÉJV¬d%+YÉ*²¬d%+YÉ*²¬d%+YEV²¬d%«ÈJV²¬d%+YÉJV²¬dYÉJV²¬"+YÉJV²Ud%+YÉJV²Ud%+YÉJV²¬d%+YÉJV¬d%+YÉJV²¬d%+YÉ*²¬d%+YEV²¬d%+YÉJV²¬d%«ÈJV²¬dYÉJV²¬"+YÉJV²¬"+YÉJV²Ud%+YÉJV²¬d%+YÉJV²¬d%+YÉJV¬d%+YÉ*²¬d%+YEV²¬d%+YEV²¬d%«ÈJV²¬dYóíòåËUUU©Tª¾¾þÂd%+YÉJVuAµ¶¶:u*&?¾÷îøäO~÷xçÏ_±bÅ§ùöÃþpû·ÿîõ¿óß×¿^¾§ý¿ìÃ+.N½sl_Á>¼xú´P]·¤ä¹Ýtßûîùzå¿)Ø·éïÿãjü÷ûðâ)%XòÞ7ÈJÖ'[yyùäädLWWWÇDû3YÅ ¶8ßöÙXÃßjÉ7XÈïo¸xxÅZü:hÇ^<±ä½o<ÿüó"ë,È2ýç?ÿùÿduÿþýyÀÝÝÝñêOú(â×ü7ÚùUSSóüÄvÈ£O>ù$þÛþþ÷¿·)´,d-**JOÇ¯r¿áÈJV²U*hY+**ÆÇÇ>z58¦ÉJV¬"ëjkk;yòdLÄekk+YÉ*²Ud]P===EEEUUU½½½d%«ÈJVuyEV²¬Y³O?ýô§?ýé_|aSäÑoûÛ_þò¶C~8q¢¯¯ÏvÈ£øoûÇ?þÑ¦Y%I"«$IdÕ¬eßx.g<^nÝ½79ÖLÍö¶ÞÞÞÆÆÆøÞb;Øtóª¿¿ÝºuÉ·ßÓÓcë¬K¦ìógÏYæÅZ]]]æ	Qsl"Û3³K.ÅDGGGmm­M7¯BÄÎÎÎmX^^në¬K¦ìógÏYæmÛ¶mhh(SÖÈö©.¿Î?~[Od]2eß8þß!kMdN[__ßÞ½mºù611±råÊØ÷bÐoë¬K¦ìó/¹3ù²æØD¶gv£££­­­ccc6]~uuu%gHµõDÖ¥QöùÜ¿|Ysl"ÛsJÃÃÃíííwîÜ±éR2è´õDÖ¥QöùÜ¿|Ysl"Û3³îîîæææ»wïÚtyTSSÓßßÿðÑ!Ö±m=uÉP~ã%wÆã/_Öi7í]uuõ3ÙtóêòåËõõõ1ZÝ¼ys2è·õDVIÈ*IÈ*IY%I"«$Id$Id$¬$U$U$²JDVIDVIÈ*IYµ»xñâúõëS©TIIÉ-[´§þëVû«ýWYØX¬MqúôéÚÚÚXOeeåk¯½6999Çe<xðLVsü63gf~yqqqÜÅÑ£GÓKÄüU«VÙùEViúúúòð ªª*ý,ÜÖÖöÔÈÇÖXMqöìÙ)48p`Ëtuu-º¬éÞ~ûíäÖóçÏÇÕ]»vù/#²JO¶ä«îÝ»ÓãããKZÖ>EÙÍÍÍ±ÃÇtgggL¯rË<y2¦ÛÛÛóøÖ²eM¦'&&Þ|óÍ¸Z]]Ì9vìØ´ÞKdÕRêÔ©S1*..Þ³gO<Ó¥çøáõõõ©Tª¶¶6Æ13=uf?iÆ°£¡¡!¾pÝºuýýýÙÃìÇ0íåþªÁÁÁM6¥_½ûv2ÿÖ­[q5f& FÓ>ì>ø ¦¦&xÖÕæÀc.[ s±ìïkÖ;vSäþ½ÿþû!âÆ3×+ÉOyrr2r_3-óâ/&&/á¾óÎ;5='¾dú^«ñ­åÞ9%²ªp§°Ì§ì7Þx#ÿÑGMy6¿xñâ]É,hÕÈî+÷W*·®_¿>÷9?ÔÉ~Ù/l¦iµó5äuÖ;ÍþY@I9^V=wîÐÒÒc÷ÈÄrgÎiãLÛ´cÖ#GÄÕµk×&sâ7¸:00cçÈªnÃÉkhh(ó	.yé¥â¹/.czóæÍståå_±Nòv]6reæ_ÅMÓ)nÞ¼9ÓõÇtàÓ===1½÷î9®vQ¶À5äq§³n´ýû÷'aÚµÅHzÕªUq¿asYfçÎeee±«Ä·vøðáÌ_òuJÇKÈqw1~MiçÈª.Å¦¥+<ùÉûyqù²á¬®Íå5À<î+³=öÄMñD¼qãÆÍ¤oºråÊÁfzÉ=f~»1Çjç.ë¬[`Êò¸ÓY7Z§cûÇ2ù-¼J~	7ÇãÌ=¬ï¢¶¶öÝwßMnºzõjÌS"«¬Ù½£vÉM3ñDe±Z ´cÇä Ùæææd~<MÇÕC%i@ò]gªå^íLNä·¦,6ß;Í£E×®]h,pâÄy-³ûöøý#Q?ð´ïÑ>ÌëÖt§O^|ñÅÜ;§DVtÉnçÎ²²²d~òziæéO$ÏwôQ<å½üòËóråÁéñ÷5chïÝ»9~JÈ¹uëÖfzlÉ±]]]s_mö¯#ùmÜ[#ÇNYs~íîÝ»qkæGHç¸Ì¶mÛbæ+¯¼ÓñKLïÜ¹sqeÇMéc£fÚ9%²ª rHò¼ùpº5^ºt)¹iÓ¦Ms98eÊÕd4íg1sÜWgáäi7]ú Ø5kÖ¤gÆåèèèU9sfÊ=¹WY~[ ÇÖËNYs~íÀ³>òõOåÇÐvqeª3ãiçÈªBï½÷Þ«¨¨H>ØpÿþýLtÔÕÕeïbÌO¬µµµéç¾Y]9uêTbmÓ²1Óåx¾sçNkkkòépqxx8ß××|eûöí7nÜH8rÊªâñÄÅw#¡w¦ßÚiµå·rl¹Üiö¦Èc£%ÑÜ²æX¦³³³¦¦&¾ñÆÆÆ.Lÿ°Yß2wÂvN¬$¬$U$²J$²JDVIÈ*IY%IY%I"«$Id$Id$¬$U$²J$²JDVIöþ/N¨4òÂ)öIEND®B`


count of sales units for 3HP/c


÷õà¶] ²,K+²·¥ï­lv±ËÒ[ÏT|e©]¬[Ià.K%B°"¯»Ms­ØÐ4õ~¯ï0f1N&:1!<óÌdæÉùæ?óÌGnÅó»PVPVPV@Y@Y@Y@YeeeJý7ç¶üëç»Ì,[¶lq²²§¬¼ ¬°ÒËºÈ«uÞ9ëµk×vïÞ½zõêT*UUUõüóÏOMMe.âÅÛ·o/¿­³³óÒ¥KÙ7qæÌ¦¦¦t:ýÄOåÞbÍ5mmmÉÊýû÷×ÔÔÄíÆµÅÕÆd_¸§§§¥¥%ÎÝ´iÓøøø¹sçãÊÛÛÛ3J®¬O=õÔ¬>÷ÜssõÃ?ôf¯¬®®ÎDnpp0û¬Ü[,++ÏIVgÝÄæÍ³/mãÆQßÌÉ(·4(+Ü×²Î'·¬Ií®Ë§Oå9#ýì³ÏÆÉmÛ¶]»-âd¬LÎ^ÆÉÈNNNNLLdU/¼ðÂÌÌL2Ó­­­5q±|ùòåXvf_xÏ=ÓÓÓÉÉXÇÉõf_PV(¹²®_¿>ëëë£§Nº~ýú|Óß¡ÆÉ¹&'#Æq²²²29¹zõê89>>î¬[Ì|mâæÍQÖÈmkkk2£Í¾p:ûäÕ«Wc9ÂìÕ_PVxeÍ¿>ytt4k"ùÖ[oÍyU©TjÖ5ÇÉX9ßíæÞbt1snLFg=·<ß®ËùOÊ¥UÖÄ»ï¾ûÒK/%ÏèÖÖÖÎy±$³æ¬±rásÖì[ljj5/¾øbOOÏÄÄ²²ÂCRÖäuÖäõÎ¿Ær:Î>kê¶8ÙÕÕ¼Î:q[á=ö$îìì;vì¸~ýúÕ«W·nÝ¿¬ÉøÂ1ýÎw¾£¬ ¬ð5Z8ë)Ù]»v%g%;;wÆÉK.%ÓìûÛßff½Ùgµµµå/k&½ÊÊÊøìi¬¬ ¬°ËóÑûöÕÔÔ$yûÒ¾tãÆä¬S§NEyêÈÈÈöíÛÓ·Å$uÖIo½õVÌtã¬ä	ÞY»ûf_8æµq±¸¨uÜhdÖ~V´ä©ã¾¾¾X>räHljjrÏ²H^gåÀîPV SSS÷î]»vmò,|õ«_u·²²ÊÊ:¯?ýéOÝRÖìêÕ«eee;Þ1¬¬%QÖ|ä#x((«²*+²¢¬Ê²*+ ¬(«²(+Ê ¬(«ßF@YQVePVeUVeEYeUV@YQVePV@YQVeeUVeUVePV@YQÖËd]]]ædÿúõëS©TKKËàà ²Ê²Þ¾¾¾¦¦¦ì­ª¡¡áüùó±ÐÝÝÝØØ¿ùÍoFîôÎ;ï¤Ói¿²¢¬³mÝºuttt¾­ª¼¼<>wuu=ãÑGõÛ(+Ê:ÏmÏµUìÞ½ÛPVµeìììò:+ ¬(ëbË:66ÖÕÕ5>>nß``%uÃÇËìîî5÷y¯Ò9×dº3ÊÊÊbMoooSSS*jnnîëëSÖR)kü`ÚÛÛãÿ¯¬ÀC_ÖX)åøÜØØ¸Ô³ÛÜ½Js×äü¿öÚk±PUUuåÊXÏsÆXYLYãýw²+¹¬¯¼òÊ[o½ËñyïÞ½É%ÇÇÇ7oÞSÃèî¥KbÍàà`kkk¬©®®©mòµX·n]¬|ûí·Þ«4ÿ~¦áòåË1d¹¾¾~ll,yê1Õ"J®¬Ñ­d_ÎøÜßßûöí===±pîÜ¹¤j1­åX¸xñâ5k¯ýæ7¿933Y¸Î÷,î7»2OY;::å|yyy>gV*«²PYãóSO=[ZZ2kÒét¦I5¯]»3Ô;wf¹ÏÕ»vqÎw1Ý¸qcöÃÃÃÃIb³×+«²PY_õÕ'NìÚµ+»¬7nÜÈ¾ddìäÉ½½½~øa¦¬óu±sÖýû÷Çð2'³'Ç³&ÊÊª¬¥RÖ±±±êêêäÕÖdÍO>ùòË/ÇBÔôñÇOZñâÅ©©©Ý»wßµ¬E³¶µµ½ûî»Ù.ÜÝUY)kØ²eK²p¦µÔ¨iKKËèèh¬9|øpEEÅ5k^ýõûPÖÌÉCæ9çäUÞj²kU,+«²VYQVePV@YQVeVBYGFF¶lÙN§ËËËÛÛÛ7´ttt,äÑY»ßÓñ"XÎ=èROOOîÀÆÇÇs÷Lõå7nÜèêêo|Ýºu½½½Êª¬Å)kccã'fnóÍ7#3óEô®û@EV#ÏK×98==½~ýúÜ<y2ªÿË÷ïßàÀø®#«ËúXNÊPZe]³fÍ¬=l3Çå?üpãÆ«W¯>sæÌ]ËÄ5yiîÁ[[[º|ùrMMMöî¾1ç_~ùåæ,²zêÔ©ü_ÞÜÜLÐ=¬¬Å,k¨ººú'xåWÎ??+1±K¶²fNæñßüfTðÖí£R¼ðÂùX^Èñ%®µBç^²¡¡¡½½=ºóËãÜIL²cÂúÞï)«²§¬!âM=tèPLOÿ(û^Òééé*kîÁÇÆÆFh,fßVT?9qo-n49dcî9r$y¥9¬¬ÊP´²fÜ¼ysõêÕ³Ê>"Îº§gs4µ¿¿?ÏîiÎz×g¾9¿<fêÙóWeUVâuÝºuÉ!Â»ï¾ÌÞb>t±³³ó¥^º|ùr®eíéé)//OÎ=8â­ÛGq'ÿfuñsÖ<+wcÎÚÞÞ>ç%÷ìÙsüøñXÊPY`±eÙ¶mÛ5kbÞÆÊ'x"êoÚ´©±±1ò¹ù¾ý®Ü#XsíÚµ»îoå¦¦¦O>ùg®¹Áttt$¯ÅfF¨¬ÊPÌgQVePVeUVeEYÖ²Æú'NðÂgÿúõëS©TKKËàà`²+PòÿÝûúú4OYX¡e4NMMÝkY#Ktww766ÞºT£äÈG±¾ªªJóUYZÖ£GîÛ·¯°uåååÙ'Ï9|UYUÖø¼iÓ¦ä(ó½£&ÏÑvïÞ,OOO¯^½:.YÍSVeVnY#«×æ¬'===ÙG8BYXqeûöí;zôè=ÍYÇÆÆºººÆÇÇs¯vY/PY eyçÿët>½½½íííñP½OÓÐÐP²Ûpö1QÖâõ7Þxì±O¶µ¦6üç¿ÿû6ÀR58qbáe­««5lnnÙêæÍçÈ¢¬*ëW¾òOÿÝ§wo?|ítz eEYSY÷ü¯mÃ¿<³?=ðØcµ eEYPVUYeEY@YUYPVUYeEYPVUYPVUYeEYPVUYUYUYeEYPVUYUYUYeEYPVUYeEYUYeEYPVUYeEY@YUYPVUYeEYPVUYPVUYeEYPVUYUYX&ëêê2'kkkS©TssóÙ³gPVõôõõ555eoUÇ×^mÇÊ(+Êz¶nÝ:::½UUUUÍÌÌÄÂÍ7¹ìë¯¿þ?î´mÛ¶U«V)+ ¬(ë<·µU¥R©YËÞy§íÛ·++ ¬(ëÊZVVYN§Óe]TY«««oÞ¼<ËÊ(+Êº¨²îÜ¹óèÑ£±;;;PVuQeíëë«©©)++«­­íïïWV@YQÖ%§¬²¢¬Ê ¬Êª¬Ê(+Êª¬²¢¬Ê(+Êª¬Ê(+Êª¬²¢¬Ê(+Êª¬Êª¬Êª¬²¢¬Ê(+Êª¬Êª¬Êª¬²¢¬Ê(+Êª¬²¢¬Êª¬²¢¬Ê(+Êª¬²¢¬Ê ¬Êª¬Ê(+Êª¬²¢¬Ê(+Êª¬Ê(+Êª¬²¢¬Ê(+Êª¬Êª¬Êª¬²¢¬Ê(+Êª¬Êª¬Êª¬²¢¬Ê(+Êª¬²¢¬Êª¬²¢¬Ê(+Êª¬²¢¬Ê ¬Êª¬Ê(+Êª¬²¢¬Ê(+Êª¬Ê(+Êª¬²¢¬Ê(+Êª¬Ê²*+ ¬(«²Ê²*+²*«²*+ ¬(«²Ê²*+ ¬(«²*+ ¬(«²Ê²*+ ¬(«²(«²*«²Ê²*+ ¬(«²Êª¬Êª¬Ê(+Ë¥¬½½½MMM©Tª¹¹¹¯¯OYeEY¥ªªêÊ+±ëêêbá>¸p§sçÎ¥Óiee½»úúú±±±XÏ±]]]äxôÑGPVõîËËËc;Ï±æw¿ûÝ¥;ýâ¿0ge]6'Ý¸q£×YeEY%JÍ¹¬¬²¢¬yêÐÐP,p!æ¯Ê(+Êº(/^ Æl5>Ç²²Ê².9eeUVeUVeUV@YQVeeUV@YQVeUV@YQVeeUV@YQVePVeUVeeUV@Yy¸ÊZYYÙÖÖ622rãÆePV[ÖG²ÔÔÔ|á_¸téÒÌÌ²(«²brrrpp°¥¥¥¢¢"»²õõõÝÝÝÊ ¬ÊZ¸.ìÜ¹3»¯qRYUYïÁÄÄDOOOsssyyyvS×­[W[[YePVe]Ôë¬/^³fff"·Ê ¬Êzªªª¶nÝjß`eâ¿Îª¬ÊJÊúÒK/ÅÌ5Y®¨¨xúé§@Yµ@Û¶mÍ¢¬¬,ûe×ÎÎNePVe-D*Íbll,9yùòå8+@YµÉ"úúúçÎKñfeQÖ#G<ãðáÃÊ ¬ÊZ Ó§O×ÕÕ¥o«­­=sæ=UYeeUVeUÖ¹téRmmmYYYöë¬7á(+²*ë½©©©ÉjÂ»nUYýÊÛúûû=ìPVPÖªªªû¼Y(+ ¬<Ìe=öll÷î½~ýº²(+Åy6x0(«²¨l.ö`PVe]6PVUYUYlhh¨±±±¢¢"yyµµµUYUYÔÓÓ3kÇ¥XèèèPVeUÖB$Ç`Î.«ÿÏ ¬ÊZèWÞ,DYgff¼ë@Y)üçZYYÝ0	j²P]]­¬Êª¬èîîÎ=RÄÉ'@Yµ@çÏ_»vm:N¥Rµµµ§O¶o0²*ë²¡¬²¢¬Ê ¬Êz/û;"¿²ÊJqÊ9vY@YµnÜ¸ñâ/ÆVrêÔ)ePVe-äHÞÏ ¬ÊZ¬öõõy@Y)òLk×®UVeUÖEíÁY½yó¦²(«².Ê(+Êª¬Êª¬xuI¡¬²ò¿Î:gPKqÈe¹¬Íâ«_ýêÔÔÔµk×yæ8ùü °k»qãFWWW:^·n]oo¯²ÊÊ+kTpÖf'ËËË»¶ýû÷8p`ff&²Z__¯¬²²BËúüóÏOLLLNN¾ðÂ)kssóððpöoûÛÿýNýìgS©²ÊÊÃYÖCå¾ÎúÚk¯vmÌW_5ÂÖ÷Þ/Ö|ïßæNûÜçV­Z¥¬²òp5<y²®®.¢ó×ÚÚÚ7ß|s1ûC9r$FFFZ[[=(++±¬E(ÿùö+VV@YQÖÚ³gÏñãÇcahhèñÇWV@YYe666VTT$ÇïYÜèèèÙjèè¨²ÊÊ+kOOÏ¬#.ÅBÔqéÆª¬²ò0µ¦¦&6áááì²ÆüUYUYúÊÛ(ëÌÌÿ| ¬þs­¬¬Í¢¿¿?	j²½¯²(«²ÞîîîÜ#E<yRYUYtþüùµk×¦ÓéT*U[[úôé%«²ÊÊC^ÖûLYeåa.k]]]CCÃ|ï=UVeUÖüçssVeâõèÑ£±YìÝ»wrrrffFY[?ë,ÞÏ ¬ÊZø³Á¹æû75Ê ¬Êjß`eeUV@YY^e]êSPVVnYïsePVUYUYUYeEYPVkYóp¤eUÖS#E(«².Ê(+Êª¬Êª¬Êª¬²¢¬Ê(+Êª¬²¢¬Êª¬²¢¬Ê(+Êª¬²¢¬Ê ¬Êª¬Ê(+Êª¬²¢¬Ê ¬Êª¬Ê(+Êª¬²¢¬Ê(+Êª¬Ê(+Êª¬²¢¬Ê(+Êª¬Êª¬Êª¬²¢¬Ê(+Êª¬²¢¬Êª¬²¢¬Ê(+Êª¬²¢¬Ê ¬Êª¬Ê(+Êª¬²¢¬Ê ¬Êª¬Ê(+Êª¬²¢¬Ê(+Êª¬Ê(+Êª¬²¢¬Ê(+Êª¬Êª¬VOOOMMYeEYïÁôôôúõë3ÚåËq§ýìgétZYeEYäå_>xð`fSëêêz$Ç£>ª¬²¢¬wwåÊÖÖÖÌ¦víÚµîôË_þÒPVuA:::Î;÷¯ò:+ ¬(kÆq'ee-ZbÍYeEY@Y)É²æ¡¬²¢¬Ê ¬Êª¬Ê(+Êª¬²¢¬Ê(+Êª¬Ê(+Êª¬²¢¬Ê(+Êª¬Êª¬Êª¬²¢¬Ê(+Êª¬Êª¬Êª¬²¢¬Ê(+Êª¬²¢¬Êª¬²¢¬Ê(+Êª¬²¢¬Ê ¬Êª¬Ê(+Êª¬²¢¬Ê(+Êª¬Ê(+Êª¬²¢¬Ê(+Êª¬Êª¬Êº?üJ:½ª­í3Åýxì±ºýèGw@YQÖWÖîÿ»/~7woq?êÿöo¼ñÇPVu%õ¯*W÷:ãcÇUVPVUYPVUYeEYPVUYPVeUVeeUV@YQVeUVPVUYPVUYeEYPVUYPVeUVeeUV@YQVeUVPVeUVeUV@YQVeeUVeeEYUYeEYPVUYeEYUYeUVeUV@YQVeeUVeeEYUYeEYPVUYeEYïYÿúõëS©TKKËàà ²*+(+Êº(çÏîîîÆÆFeUVPVµhÊËËãó·¾õ­ÏÞiË-1©UÖÂ|ðÁ_YÛ¶mÛ³gOq¯3®0®v)Fûþûï4GYYqeØ½w,|ÿûßß§§~zÕªUÊZJüÇ=PÜt:õé¿ûTq¯ó?6Ö-ÅPã:úÓz4GYYYeìììòlðRµîSô¡ÖüÍ_ûîÞâ^çÿÞ·£ù?­+úP?³±YYQVVVYÇÆÆºººÆÇÇí¬¬Ê²¢¬ÕÛÛÛÞÞíÌseUVeEYQÖª«û×Ö2UYeEY²*«²¢¬(«²*«²²*«²*«²²¢¬Êª¬Ê²¢¬Êª¬Ê²¢¬Êª¬ÊÊ²*«²*+Ê²*«²*+Ê²*«²*+(«²*«²*+(+Êª¬Êª¬(+Êª¬Êª¬ ¬Êª¬Êª¬ ¬(«²*«²¢¬(«²*«²¢¬(«²*«²²¢¬Êª¬ ¬(«²*«²¢¬(«²*«²²*«²*«²²¢¬Êª¬Ê²¢¬Êª¬Ê²¢¬Êª¬ÊÊ²*«²*+Ê²*«²*+Ê²*«²*+(«²*kéuó¦õÏ<óÌEõõ¯½¦æ¯WrYÿý¿üå7íg?ûB,o,wÞyGYQÖePÖµU´<3×"~üÖOU~üc+¹¬ùÛu5Å½W?úÑüã?þh-	bqúñ×,Ê².²6üOøÚ3Ëb¨Ë¨¬K1Ôø1þó­åRÖ¢oTñ¢¬(«²*«²*«²¢¬Êª¬Ê²¢¬Êª¬Ê²*«²*«²*«²*«²¢¬Êª¬Ê²¢¬Êª¬Ê²*«²*«²*«²*«²¢¬Êª¬Ê²¢¬Êª¬Ê²¢¬Êª¬Ê²*+Êª¬Êª¬Êª¬(«²*«²¢¬(«²*«²¢¬Êª¬Êª¬Êª¬Êª¬(«²*«²¢¬(«²*«²¢¬(«²*«²*«²*+Êª¬Êª¬Êª¬(«²*«²¢¬(«²*«²¢¬Êª¬Êª¬Êª¬Êª¬(«²*«²¢¬(«²*«²¢¬Êª¬Êª¬Êª¬Êª¬(«²*«²¢¬(«²*«²¢¬(«²*«²¢¬Ê²*«²*«²*+oYkkkS©TssóÙ³gUYeEY¥³³óØ±c±ðÚk¯íØ±#~õ«_½s§3gÎ¬Zµê_õå/ùÉøôþùøñµÿótzUª¸×øDÕ®®Ï.¡~üãû§ÿgq¯óéÏÿ·OÔüõ²jü::úÈXÑ7ªxHÅIu	UUUÍÌÌÄÂÍ7ëêêb¡««ë1©MêÑGkøwÅ¶D×¹ºtßþRµ¬¬,¿Äö?onCß¸ÒmTù6?ö±².¡ø=µü?üáÿå¸~ýzÑoztt4~=ÞÿýÒÿi?>÷CéõÇ?þqõÏþséõÈ#Ëâ×õÀËb¨1kkk[Cýâ¿ØÑÑ!<e¿Ä3Ëñ§Üý¼ieUVeUV1à!,kuuõÍ7oÝ~68UYUYe];w=z4âsgg§²*«²*«²¢¬Ò××WSSSVVV[[Ûßß¯¬Êª¬Êª¬(ër¥¬Êª¬Êêe-¦ßÿþ÷ßøÆ7®^½ZúCýõ¯CýãÿXúC½xñbõ/ùKéõÂZÛêÀÀ@ü°,úóÿü?üá²êO~òS§Ny$DY@Y@Yb¹G*^È±ÔÕ«WãOÍ7Â|ÿúõëã¶ZZZâvKy¨CCC6lHn«¯¯¯èééÉñ®d:>>d´Rê7ºººÒéôºuëzK@Y·Ü#ç®¹âq¿©©)û8¢yÆó`ßÐÐpþüùXèîînll,å¡ÆbòZ¸ªªª¦§§ãOÌ6P²C=yòdä*ÿxJd¨û÷ï?pàÀÌÌLdµ¾¾¾Ä7uÙË=RqîûiëÖ­É^ÊyFX///_C=sæLÌ°K|¨/¿üòÁ3Û@É5²:kjüi5<<¼,²>rT»æü<²Êg<%2øÝ»wøPc"¸zõê¸cc]ÊC½råJkkk<g¶jCCCÜJxdd¤×ÿê«¯Æß1aï½÷Å¯Êºå©ø»xÎ²æO)~rr²³³sjjªôzëöëÉQ3Kv¨çÎËÞJÿ^K	(Ù¡Æm%ïS¿âïeq¯¢¬ËXîà±ç,kñ<ðÁÇCjWW×øøxéuÖ£d:ë&.5éMÉ5ûúK|@Y¹G*~Ç.³¬yÆó`ßÛÛÛÞÞljCCÃÐÐÐ­Ûû3ÇKy¨¹Û@)ß«.]JþÀ*ñuÏ=Ç¿u/ñÇ|Yl(ëòüo?rå©ø»xÎ²Î9R|]]Ý¬ÙUÉupp°¹¹9&+7oNfØ%;ÔÜm d:00ÐÔÔ÷ê¦M"®¥<Ôä%áÑÑÑe± ¬²²²²ÊÊÊ(+(+(+(«»Ú¹sç6nÜJ¥ÊËË·lÙ²¨­ùßþ«ÝûuZÜuW¼òÊ+õõõq=555û÷ïÏ?¼ìÙÿ0NÇ<x0sÉX_YYi£eå>( µµµGó;w>4e-àÞ(Ê]YäNÏ?ÿ|eÍxõÕWsÏ9'·oßnSe¥¤glÉW]»v-oÞ¼¹¬ËºÈÁå®ØºukLyO>Ëï¾ûn¼§²&ËÓÓÓI¤ëêê5ûöí³©²rëØ±c1J§Ó»víGÌÌú7ß|³¹¹9J566¾õÖ[óU!÷Á7¦/---ñ6lÊîäaÎÛÊÿU###6mÊ<;úá&ë¯'ce2Î9ìãÇ744Äeb¨1à»^m..äÈ¾Xî÷u×ó®ÈÿúÁ~°zõê¶¶¶ù~î===q±øÚÊY·,á_1¤ü ¬¿x(Ì~ÈþÚ×¾¬ûí·g=;wn]Éi¹k#ç»­ü_QÉ>wãÆÉú¸Åìõs#J¶Lç»Ú*kî=¿¬w½ÑÜ/¹ë(1ßÓ³qqnMMMÌù¬~ÖõÀq²¾¾>YIÄÉááá< ¬+Âã?Ì±FGG³(cæ'öÙxÏ±¼yóævå¹çYT²«K)Ñ½ÞV¶²²²8kÎ9eâòåËó ®?#N±Ü××Ë;vìXàÕåuÜè]ï´½÷&wÂ×ÓÙ¸LEEEr',¼¬³:t(Îo6¾¿ÆBPÖ!çLWô Ö'¯çÅç</Èå>øNMMÝµ%ÝV¶]»vÅYñÞÖÖ³¢ÌY1áZ[[ç@rÙ¢4w½Úõ®÷À¬k(àFïz§åïtô8	Ì5óÜÕyönllüÎw¾õÞïÅÊ¸ÃóoT²® ²&Sj5_0´¬ÑÐ¶mÛfÛÛÛõñp'_zé¥dÂ4çï:[T-ÿÕÎ×ÂîY»×-øNê©§âoL¹³_(-àuÖ'NÄYÏ<óLþPÖ!yâîôéÓÃÃÃ±°fÍdò|iöw$o¿ýv<t>÷Üs÷Ô7ndæsynk!ûÐ^»v-»Ir®röìÙùÆ¼ÛÓÓ³ð«Íýs¤° ÿ½çFg]sawÚO<géK_å£G.r¦ç>Îzýõ×óoT²®³v6É¼»1yª0Ûùóç³6mÚ´¬¬¬ï½yn+Ï£yòðÙ	vÝºuñyrrrÖU<yrÖ-nÝº5ÿÕf+ìÈso,äFg]sawZÿ¬¯:~üøâËSáì¿TæÛ¨e])>]¼AâúõëÙÑMÞ:ÒÔÔ=½9_YYYLw2¡wíÊ±cÇjjjâÚæÌÆ|·çÑ|||¼³³3ywMtqll,Y?00¼åÉ'¼téRæM³®*Æ·ßEÌ¨bÞyt¾«ÍVØ=çÞXÈæÞÜi·nÒ!¯ÊdueMþÉÞxæÛ¨eeeePVPVPVPV@Y@Y@YeeenÝúÿ_»ËÑ¼ùIEND®B`
