# Supplementary material for: Statistical analysis of the count and profitability of air conditioners
Source: Data Brief. 2018 May 15;19:413–23. doi: 10.1016/j.dib.2018.05.035 (PMC5997838; doi:10.1016/j.dib.2018.05.035)
Supplement: Supplementary file 2 — Supplementary material [file mmc1.docx]

**Conflict of Interest**

All the authors confirm no conflict of Interest.
